# Suicidal Ideation in Online Spaces Through the Lens of Interpersonal Theory of Suicide: Exploratory Study of Self-Disclosure, Peer Support, and AI Responses

**DOI:** 10.2196/86265

**Published:** 2026-06-03

**Authors:** Soorya Ram Shimgekar, Violeta J Rodriguez, Paul A Bloom, Dong Whi Yoo, Koustuv Saha

**Affiliations:** 1 Siebel School of Computing and Data Science, Grainger College of Engineering, University of Illinois Urbana-Champaign, 201 N. Goodwin Ave, Urbana, IL, 61801, United States, 1 2172443824; 2 Department of Psychology, University of Illinois Urbana-Champaign, Champaign, IL, United States; 3 New York State Psychiatric Institute, Columbia University Irving Medical Center, New York City, NY, United States; 4 Department of Human-Centered Computing, Luddy School of Informatics, Computing, and Engineering, Indiana University Indianapolis, Indianapolis, IN, United States

**Keywords:** suicidal ideation, interpersonal theory of suicide, online mental health, Reddit, natural language processing, psycholinguistic analysis, artificial intelligence, AI chatbots, digital mental health interventions

## Abstract

**Background:**

Suicide is a critical global public health issue, with millions experiencing suicidal ideation (SI) each year. Global estimates suggest that the lifetime prevalence of SI ranges between 9% and 12% worldwide, underscoring the scale of this public health concern. Online platforms, such as Reddit, provide spaces where individuals express suicidal thoughts and seek peer support. While prior computational research has leveraged machine learning and natural language analysis to detect SI, much of it lacks grounding in psychological theory, limiting interpretability and intervention design.

**Objective:**

This study applied the Interpersonal Theory of Suicide (IPTS) to understand the underlying psychosocial mechanisms driving high-risk suicidal intent in online spaces, analyze linguistic expressions of SI, and assess the role of artificial intelligence (AI) systems in providing supportive responses.

**Methods:**

We analyzed 59,607 posts from Reddit’s r/SuicideWatch community. Posts were categorized into 4 SI dimensions (ie, loneliness, lack of reciprocal love, self-hate, and liability) and 3 IPTS-based risk factors (ie, thwarted belongingness, perceived burdensomeness, and acquired capability for suicide). High-risk posts were operationalized based on the language markers of suicidal planning, attempts, and explicit intent. We further conducted psycholinguistic and content analyses of supportive responses and evaluated AI chatbot–generated replies for structural coherence and empathy.

**Results:**

High-risk SI posts contained frequent references to planning and attempts, methods and tools, and expressions of weakness and pain, patterns that are consistent with theoretical expectations regarding the progression of suicidal capability. Supportive peer responses varied significantly across SI stages (*P*<.001), with deeper empathy and self-disclosure emerging in replies to high-risk posts. Compared with online community responses, AI-generated replies showed higher semantic similarity (Cohen *d*=0.20) and linguistic style accommodation (Cohen *d*=0.08), but substantially lower diversity (Cohen *d*=−0.31); empathy differences were minimal in the most context-rich prompting condition. Expert evaluators further noted that AI responses often lacked contextual personalization and emotional depth.

**Conclusions:**

Grounding computational analysis in IPTS provides richer theoretical insight into SI expressed online. While AI-based systems can enhance the structural and linguistic quality of supportive messages, they currently lack the nuanced empathy and contextual awareness needed for effective mental health support. These findings highlight the need for theory-driven, human-AI collaborative frameworks in suicide prevention research and interventions.

## Introduction

Amid the escalating mental health concerns worldwide, suicide has emerged as a major public health crisis, claiming approximately 700,000 deaths each year, with a disproportionate impact on young adults and marginalized communities [1]. Beyond fatalities, millions more experience suicidal ideation (SI) or attempts, further exacerbating the mental health burden worldwide [2]. In this context, it is critical to find safe spaces for individuals to express SI and receive timely and effective support and intervention. The widespread adoption of the internet and digital tools has facilitated the prevalence of dedicated online spaces where individuals can share mental health struggles and seek peer support. Such online support tools can be based on human-human interactions, such as instant messaging and social media platforms [3,4], or human-artificial intelligence (AI) interactions, including chatbots [5-7]. Given socioeconomic disparities, limited access to mental health services, and pervasive stigma, online support tools are especially advantageous in offering several key benefits, including anonymity, peer support, and the flexibility of asynchronous participation [8]. Prior work noted how these online communities (OCs) can foster social support, empathy, and connection [8-12]. Additionally, we are seeing the rise of generative AI-driven chatbots, which can provide immediate, AI-driven, human-like responses to mental health queries [13-16]. Despite these developments, a critical gap remains in understanding the theoretical foundations of online interactions in high-stakes contexts, particularly SI.

Prior work has highlighted the effectiveness of OCs, such as on Reddit, TalkLife, and 7cups, in providing spaces where individuals can discuss, seek, and share information, advice, and social support related to mental health concerns [8,17-20]. These platforms, with features of anonymity (or pseudonymity), moderation, and structured peer-support interactions, create safe spaces that encourage candid and sensitive self-disclosures, promoting a sense of belonging and solidarity among peer supporters. Earlier research highlights that participation in moderated peer-support spaces can improve mental well-being [12,21,22]. Relatedly, De Choudhury and Kiciman [23] examined the language of social support in response to SI in OCs.

Despite advancements in detecting SI in online spaces [24-27], a critical gap remains in applying a theoretical lens to understand the mechanisms underlying these interactions. Given that suicidal thoughts rarely emerge in isolation, a more nuanced approach is needed, one that accounts for the psychological and social factors influencing suicidal progression [28]. The Interpersonal Theory of Suicide (IPTS) provides a well-tested framework for understanding the mechanisms of both SI and transitions from ideation to suicidal behaviors (eg, “ideation-to action” [29]), shedding light on the different dimensions and risk factors of SI [30]. IPTS posits that suicidal behavior is most likely when 3 key psychological risk factors converge: thwarted belongingness, perceived burdensomeness, and acquired capability for suicide [30]. Given the growing reliance on OCs for mental health support, applying this theory to online discourse could enhance our understanding of suicide risk assessment and intervention strategies in digital settings.

While several contemporary theories model the progression of suicidal thoughts and behaviors, including the three-step theory (3ST) and the integrated motivational-volitional (IMV) model, we selected IPTS as the primary framework for this study because of its strong emphasis on interpersonal and relational constructs. Online mental health communities are inherently social environments, where expressions of belongingness, burdensomeness, and perceived social disconnection are frequently negotiated through language and peer interaction. Compared to 3ST, which emphasizes psychological pain and hopelessness alongside connectedness, and IMV, which highlights motivational and volitional moderators such as defeat and entrapment, IPTS offers a more direct mapping between interpersonal perceptions and suicide risk. This makes IPTS particularly well-suited for analyzing discourse in online support settings, where social dynamics and relational self-concepts are central. Our findings in the *Discussion* section further show that patterns observed in online language align not only with IPTS but also with broader ideation-to-action frameworks, reinforcing the value of situating IPTS within the larger theoretical landscape of suicide research.

It is essential to provide immediate, around-the-clock, and portable assistance to individuals experiencing SI. A plausible solution to this could be AI chatbots, which provide spaces for personal and interactive journaling, as well as educational resources for self-help and coping strategies [31-34]. Although such tools hold promise for supplementing traditional therapy, concerns have been raised regarding their effectiveness and the importance of maintaining human oversight in mental health care [35]. Furthermore, effective response strategies for different types of SI, in terms of linguistic characteristics, remain underexplored. Such an understanding can help improve timely and tailored online mental health interventions.

With the abovementioned motivation, this paper has the following research aims:

- Aim 1: to examine how SI manifests in online self-disclosures through the lens of the IPTS.
- Aim 2: to analyze what linguistic cues are associated with responses to SI disclosures in online spaces.
- Aim 3: to evaluate the language of an AI chatbot’s responses to online SI disclosures.

We conducted our study on 59,607 posts and 149,144 comments collected from the *r/SuicideWatch* subreddit on Reddit, an OC dedicated to SI-related discussions with more than 516,000 members (as of February 2025). First, for aim 1, we adopted a theory-driven lens of operationalizing IPTS within our dataset using unsupervised machine learning and iterative codebook development. This approach allowed us to label the approximately 59,000 posts based on dimensions (loneliness, lack of reciprocal love, self-hate, and liability), as well as risk factors (thwarted belongingness, perceived burdensomeness, and acquired capability for suicide), using a data-driven process informed by iterative codebook development.

Next, for aim 2, we analyzed responses to SI posts through psycholinguistic analyses (using the Linguistic Inquiry and Word Count [LIWC] [35]) and content analyses (using Sparse Additive Generative Model [SAGE] [36]), identifying key linguistic characteristics of responses to different kinds of SI posts.

Finally, for aim 3, we explored various prompting strategies for AI chatbots (using GPT-4o), incorporating risk factors and key characteristics of supportive responses. We compared the AI-generated responses to human-written responses in OCs, finding that although AI-generated responses consisted of better linguistic structure and semantic alignment to the original post, these responses were less diverse, more complex, and more formal compared to human-written responses. We also expert-validated these AI responses with our psychologist coauthors to identify persistent limitations of providing genuine empathy.

This paper makes the following key contributions: (1) a theory-driven computational framework to label online disclosures of SI, (2) a linguistic analysis of supportive responses to various SI posts, and (3) a preliminary evaluation of AI online disclosures of SI posts through quantitative analysis and expert evaluation. This study underscored the value of incorporating a theory-based lens in building digital mental health interventions, demonstrating how established psychological frameworks can enhance suicide risk assessment and support strategies. We discussed the implications of responsible design and deployment of mental health interventions. For instance, online platforms can integrate IPTS-based models to assess and triage critical cases of suicidal risk for timely intervention. Although AI-driven mental health support holds potential, its effectiveness hinges on its adaptability to individual needs and the presence of human oversight. Rather than functioning autonomously, AI should serve as a complementary tool that enhances, rather than replaces, human-led crisis interventions [37-39].

## Methods

### Data

We sourced our data on SI from the subreddit *r/SuicideWatch* on Reddit. Reddit is a widely used semianonymous social platform consisting of OCs, called subreddits, which are dedicated to specific themes of discussion and topics. Prior work has obtained and studied Reddit data for SI [23,40] as well as other mental health concerns [8,17,21]. This body of research established that design features such as pseudonymity, community-driven moderation, and asynchronous peer support on Reddit enable individuals to overcome mental health–related stigma and candidly self-disclose their sensitive mental health concerns and seek social support from other community members [8,10,17,41]. Essentially, Reddit has several communities dedicated to mental health discussions [17,21], and among these, the subreddit *r/SuicideWatch* self-describes itself as “peer support for anyone struggling with suicidal thoughts.” The subreddit was started on December 16, 2008, and had more than 516,000 members as of February 2025. This subreddit is heavily moderated with 8 active moderators, and the community guidelines explicitly prohibit harmful responses, tough love, guilt-tripping, and actions such as trolling or promoting suicide. Furthermore, the community advises against recommending specific therapies, self-help strategies, or medications.

We collected the Reddit data using a publicly available Reddit application programming interface (API) from *pullpush.io*, a freely accessible clone of the PushShift API. This API facilitated the retrieval of Reddit posts, comments, and associated upvote and downvote counts, along with metadata for each post or comment, including timestamps, post, comment, or user identifiers. The dataset comprises discussion threads from *r/SuicideWatch* spanning the period from May 2023 to February 2024. To ensure data integrity and respect user privacy, duplicate entries were removed, and any posts deleted by the user or removed by moderators were excluded. In conclusion, our dataset includes 59,607 posts and 149,144 comments, averaging 2.50 comments per post. It contains 59,408 unique Reddit users who participated in *r/SuicideWatch* by either posting or commenting. Table 1 presents the descriptive statistics of our dataset.

**Table 1.** Descriptive statistics of our dataset from r/SuicideWatch.

Measure	Values
Posts, n	59,607
Users posting, n	36,879
Post length (words)	
Mean (SD)	150.71 (171.69)
Median	94
Comments, n	1,49,144
Users commenting, n	37,751
Comments per post, mean (SD)	2.50 (8.69)
Comment length (words)	
Mean (SD)	22.43 (38.18)
Median	11
Posts with zero comments, n (%)	11,642 (19.5)

### Aim 1: Theory-Driven Characterization of SI

#### Overview

To address aim 1, our study investigated how SI manifests in OCs through the lens of the IPTS [30,42]. IPTS states that lethally suicidal behavior arises from the intersection of three risk factors: (1) thwarted belongingness, (2) perceived burdensomeness, and (3) acquired capability for suicide. Given its established relevance in understanding suicide risk, IPTS served as a theoretical framework in our work for categorizing expressions of SI in the online community of *r/SuicideWatch* on Reddit.

To operationalize IPTS, we used a 2-step approach where first we identified the presence of dimensions (loneliness, lack of reciprocal love, self-hate, and liability) in the posts and then, based on the intersection of dimensions, labeled posts into the 3 risk factors (thwarted belongingness, perceived burdensomeness, and acquired capability of suicide). Finally, from the intersection of risk factors, we identified lethally suicidal posts; posts with the combination of all IPTS factors theorized to confer high risk of suicide attempts [30].

Our classification approach consisted of three major steps: (1) we classified posts into dimensions using supervised learning trained on datasets resembling each dimension, (2) we developed an iterative codebook-based similarities to refine the classification of posts into dimensions, and (3) we adopted a threshold-based intersection approach to label posts into risk factors. This approach also enabled us to label posts exhibiting lethally SI. We elaborate on our approach and validation in this section.

#### Classifying Posts Into IPTS Types

**Figure 1.**
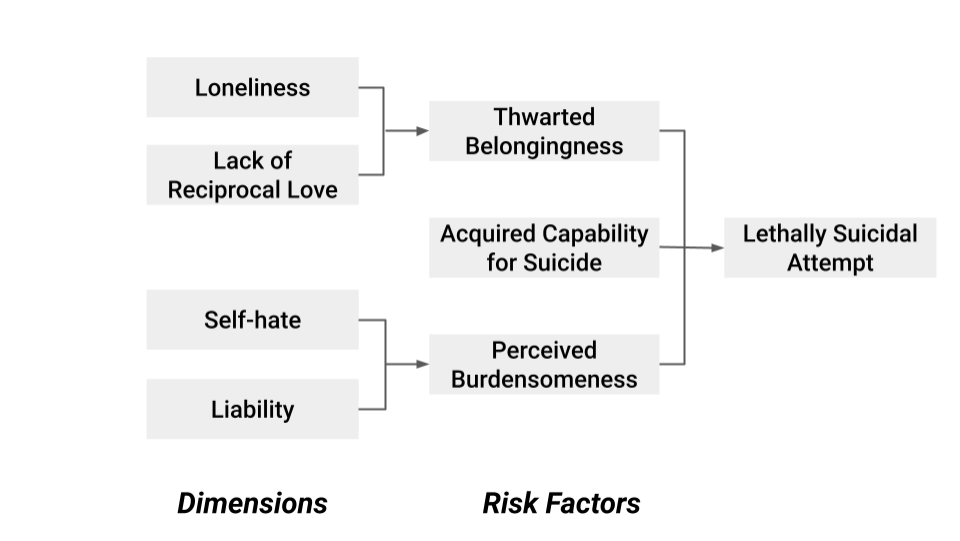
Schematic of the Interpersonal Theory of Suicide (IPTS) pathway to lethally suicidal behavior [30]. Arrows illustrate the conceptual progression from IPTS dimensions to the 3 core risk factors: thwarted belongingness, perceived burdensomeness, and acquired capability for suicide. Risk factors are then converged to enable transition to suicidal behavior.

To classify posts into the risk factors and dimensions as per IPTS, we adopted an iterative codebook-based similarity approach, as shown in Figure S5 in Multimedia Appendix 1. This process was designed to systematically identify and categorize SI expressions using a combination of distant supervision, semantic similarity matching, and iterative codebook refinement. We began by classifying the dimensions and acquired capability for suicide in IPTS, following the causal pathway in Figure 1.

##### Distant Supervision–Based Dimension Classification and Identifying Seed Keywords

First, we developed a distantly supervised binary classifier for each IPTS dimension. These classifiers were trained using relevant distant supervision datasets, as outlined in the following paragraphs. The training process involved a 55:45 train-test split ratio. We adopted this slightly larger test proportion to obtain a more conservative and stable estimate of generalization performance, particularly given the moderate size and varying class distributions of the distant supervision datasets. As these classifiers were used to generate weak supervisory signals rather than to optimize predictive performance for deployment, we prioritized robust evaluation over maximizing training data size. We trained each classifier model as a sequential neural network for binary text classification. This included an embedding layer that converted words into 64-dimensional vectors. The model then included a long short-term memory layer with 128 units, using dropout and recurrent dropout for regularization. A Global Max Pooling layer follows to capture the most significant features of the sequence. The model also has a dense layer with 64 units and rectified linear unit activation, followed by a sigmoid output layer used for binary classification. It was compiled with binary cross-entropy loss and the Adam optimizer, with accuracy as the evaluation metric. To address class imbalance in the distant supervision datasets, we applied class weighting during training, assigning higher weights to minority classes within the loss function. This approach reduces bias toward majority labels and improves the model’s sensitivity to less frequent but theoretically important expressions of IPTS dimensions.

For loneliness, we used the expert-annotated lonely dataset [43], which comprises 5633 text entries labeled as either “lonely” or “not lonely.” This dataset was selected due to its semantic alignment with loneliness. For instance, the sentence “I wish I could talk with someone” is categorized as “lonely,” whereas “I had a good conversation with dad” is classified as “not lonely.”

For lack of reciprocal love, we used the psychosocial-mental-health-analysis dataset [44]. This dataset consists of posts classified into 30 categories, of which we focus on relationships (37%), family (8%), interpersonal conflicts (2%), divorce (1%), and marital issues (1%). An example entry from the “relationship” category is, “I experience feelings of loneliness, confusion, and mood swings, struggling to find fulfillment even when spending time alone,” aligning directly with a lack of reciprocal love.

For self-hate, we used a validated hate speech dataset, measuring-hate-speech [45]. This dataset comprises 39,565 comments annotated by 7912 annotators, resulting in 135,556 annotated instances. This dataset tags comments with a hate speech score, which we used for our study. Although hate speech is not directly linked to self-hate, it contains keywords and key phrases associated with the broader theme of hate. The classification captures these patterns, providing a foundation for initiating the iterative codebook algorithm on our dataset.

For liability, we used the LoST dataset [46], which contains 3252 text entries labeled as 0 (no low self-esteem or self-liability issues) or 1 (presence of such issues). This dataset is especially relevant to identify liability, based on prior work that texts reflecting low self-esteem often convey perceptions of being a liability [47].

Accordingly, we constructed a comprehensive codebook of seed keywords and phrases (Table 2) that capture the language associated with each dimension and acquired the capability for suicide. These key phrases are directly referred from the prior work [30,42].

**Table 2.** A codebook of seed keywords for each secondary dimension and risk factors of suicidal ideation as per Interpersonal Theory of Suicide (IPTS).

IPTS	Seed keywords
Risk factor: thwarted belongingness	
* *Loneliness	disconnected, loneliness, pulling together, no care, seasonal variation, reductions in social interactions, marriage, no children and friends, living alone, and no social supports
Lack of reciprocal love	lack love, no love, social withdrawal, low openness, single jail cell, domestic violence, childhood abuse, and familial discord
Risk factor: perceived burdensomeness	
Self-hate	I hate myself, I am useless, low self-esteem, self-blame, shame, and mental state of agitation
Liability	my death is worth more than my life, distress from homelessness, distress from incarceration, distress from unemployment, distress from physical illness, expendability, unwanted, and belief of burden on family
Risk factor: acquired capability for suicide	
Acquired capability for suicide	increased physical pain tolerance, reduced fear of death, habituation, physical pain, acquired capability, lowered fear of death, past serious ideation, non-zero degree of fearlessness, courage and the ability to commit suicide, elevated physical pain tolerance, recent suicidal behavior, serious levels of suicidal intent, cutting one’s wrists, pulling the trigger on a gun, jumping off a building, and overdose

##### Labeling Posts With IPTS Dimensions and Risk Factors

We used a semantic similarity approach, wherein each classified post from the earlier distantly supervised model was compared against the seed key phrases in the codebook (Table 2). Both posts and key phrases were embedded into a 384-dimensional vector space using a transformer-based language model of MiniLM [48]. We computed cosine similarity scores between the embedded representations of posts and dimension’s codebook phrases, labeling a post under a specific dimension if the similarity exceeded a threshold of 0.60. In Multimedia Appendix 1, Section S10, we mention the process of thresholding to categorize the posts into risk factors in detail.

The labeling process was refined by iteratively updating the codebook. We used a rapid automatic keyword extraction (RAKE) [49] method to extract key phrases from the previously labeled posts and incorporated them into the codebook. We then reapplied the semantic similarity approach, repeating this process of extracting keywords and updating the codebook until the codebook remained constant. This iterative refinement ensured broader coverage of language patterns commonly associated with SI within the IPTS framework.

To prevent semantic drift during this iterative expansion, newly extracted keywords were not added automatically. Instead, each candidate phrase was manually reviewed by our psychologist coauthors. Keywords were retained only if their semantic meaning was clearly aligned with the theoretical definitions of the corresponding IPTS dimensions. This manual vetting step ensured that the evolving codebook remained conceptually grounded in theory rather than being driven solely by distributional similarity. In particular, for the self-hate dimension, we recognize that the distant supervision hate speech corpus was originally designed to detect outward-directed aggression, which is conceptually distinct from internalized self-directed negative evaluation. Accordingly, the corpus was used only as an initial weak lexical signal to surface broadly negative self-referential language, not as a direct proxy for self-hate. During codebook refinement, expert reviewers explicitly removed phrases reflecting outward hostility or general interpersonal toxicity, retaining only language consistent with theoretical definitions of internalized self-devaluation and worthlessness within the IPTS framework.

Upon labeling all the posts into dimensions, we identified risk factors by detecting posts containing the 2 associated dimensions with a given risk factor (Figure 1). To derive IPTS risk factor labels from dimension-level signals, we combined cosine similarity scores in a structured manner. Each post first received cosine similarity scores with respect to the seed phrase sets representing individual dimensions (eg, loneliness, lack of reciprocal love, self-hate, and liability). For risk factors defined by the co-occurrence of 2 dimensions, we computed the average of the corresponding similarity scores. Specifically, for post i, the risk factor score was calculated as follows:


$$RiskFactorScore_{i}={(S}_{D1,i}+S_{D2,i})/2$$

where $S_{D1,i}$ and $S_{D2,i}$ denote the cosine similarity between post i and the codebook phrase sets for the 2 dimensions associated with that risk factor (eg, thwarted belongingness=loneliness+lack of reciprocal love; perceived burdensomeness=self-hate+liability). A post was assigned the corresponding risk factor label if this averaged score exceeded the same threshold used for dimension labeling (0.60). In contrast, acquired capability for suicide was operationalized independently rather than as a combination of 2 dimensions; posts were labeled directly based on cosine similarity with their dedicated codebook phrase set using the same threshold criterion.

##### Topic Modeling Using BERTopic

To characterize thematic patterns in the language associated with SI across IPTS-informed risk factors and dimensions, we conducted topic modeling using BERTopic [50], an approach previously applied in social media and mental health research [51]. Prior to modeling, the corpus was preprocessed to improve semantic consistency and reduce noise. Specifically, we removed standard English stop words and retained posts as full text documents. For vectorization within BERTopic, we used a bag-of-words representation with CountVectorizer (stop_words=“english”). For semantic embeddings, we used a pretrained sentence transformer model (all-MiniLM-L6-v2) to generate dense document representations for each post.

We fit BERTopic models using sentence transformer embeddings and a class-based term frequency-inverse document frequency formulation (ClassTfidfTransformer(reduce_frequent_words=True)), which reduces the influence of overly frequent terms and improves topic interpretability. We additionally used a topic representation refinement strategy based on maximal marginal relevance (MaximalMarginalRelevance(diversity=0.2)) to improve diversity among the top keywords per topic. For each model, we used top_n_words=200 to retrieve representative keywords for interpretation and subsequent labeling. After fitting the initial BERTopic model, we applied topic reduction (reduce_topics) and systematically varied the number of reduced topics (k) from 5 to 14 to evaluate topic quality.

### Aim 2: Analyzing Language of Responses to SI in Online Spaces

From aim 1, we gained a deeper understanding of the nuanced characteristics that define SI posts. Now, in this section, we aimed to explore how community members respond to such SI posts. For this purpose, we examined the language of responses (comments) to SI posts using (1) the psycholinguistic lexicon LIWC [52,53], particularly the LIWC-2015 version [53]; and (2) content analysis through the SAGE [36].

Although LIWC contains more than 90 linguistic and psychological categories, we focused on a theory-driven subset most relevant to emotional expression, cognitive processing, social orientation, and distress-related communication, as established in prior suicide and mental health language research [54-56]. This targeted selection improves interpretability while minimizing noise from categories that are not theoretically meaningful in the context of SI. Statistical significance for LIWC-based comparisons was evaluated using a Bonferroni-corrected threshold to account for multiple category-level tests. This dual approach of linguistic analysis and keyword differentiation provided a robust framework for understanding the nuanced language and psychological profiles associated with the responses to each risk factor.

To complement LIWC’s theory-driven categorical analysis, we used the SAGE to identify lexical features that statistically distinguish responses across SI risk factor groups. SAGE is a generative, log-linear language modeling approach that estimates differences between word distributions in 2 corpora while enforcing sparsity, enabling it to highlight the most discriminative terms without overemphasizing extremely common or rare words. Rather than relying on raw frequency differences, SAGE models deviations from a background distribution and assigns each term a weight (η) reflecting how strongly it characterizes one group relative to another, with positive values indicating overrepresentation in the target group and negative values indicating association with the comparison group. In our implementation, responses were vectorized using unigram and bigram features with standard tokenization, lowercasing, and stop-word filtering. For each comparison, we constructed vocabularies for the target and contrast groups, aligned them into a shared feature space, computed term frequency distributions, and estimated SAGE parameters to obtain ranked lists of distinguishing words and phrases for each risk factor. As SAGE captures consistent distributional differences rather than isolated keywords, it provides a robust method for identifying thematic and stylistic tendencies in supportive responses, complementing LIWC’s psychologically grounded category-based analysis.

### Aim 3: Evaluating AI’s Responses to SI

#### Overview

The advancements in large language models (LLMs) have enabled high-quality, natural language responses to user queries. AI chatbots present a potential approach for delivering timely and effective supportive responses to posts on SI. Therefore, for aim 3, we examined how an AI would respond to online mental health queries on SI. We explored whether prompting with linguistic cues of supportiveness could enhance the quality of the AI chatbot’s responses.

#### Generating AI Responses

We prompted a state-of-the-art LLM as our AI chatbot, GPT-4o, with varying levels of context (including IPTS categories), and conducted a linguistic comparison on lexico-semantic attributes. We conducted our analyses by prompting with 3 kinds of context settings (Table S1 in Multimedia Appendix 1 provides the full prompts):

- AI-1: prompting only posts—In this setting, we prompted the Reddit post. This is more of a baseline scenario of AI responses in terms of how the model interprets and responds to SI posts.
- AI-2: prompting posts and IPTS category—In this setting, we prompted the post along with its IPTS category based on our classification of the SI post (from aim 1).
- AI-3: prompting posts, IPTS category, and linguistic characteristics—In this setting, we prompted the AI with the post, IPTS category, as well as key features of supportive responses as per prior literature [22].

These characteristics include that the response should be (1) semantically similar and linguistically accommodating to the query, (2) diverse, (3) empathetic, and (4) promoting hopefulness. Our tiered approach of prompting was aimed at offering a systematic evaluation of how contextual enrichment influenced the response quality of the AI chatbot. We obtained a random sample of 2000 posts from our dataset, where 500 posts exhibited each of the 3 risk factors and 500 exhibited lethally SI, and then prompted these posts to GPT-4o using the above settings of prompts.

### Ethical Considerations

This study analyzed publicly accessible social media discussions on Reddit and did not involve direct interaction with individuals. As the work relied on publicly available data and did not constitute human subjects research involving direct intervention or interaction with participants, it did not require institutional ethics board approval. Despite the use of public data, we are committed to conducting this research ethically and implemented safeguards to protect user privacy and confidentiality. We did not collect or report personally identifying information, and we presented only paraphrased quotes to reduce traceability while still providing adequate contextual grounding for readers. Furthermore, we ensured that no figures, screenshots, or supplementary materials contain identifying information of individual Reddit users.

As this study relied exclusively on retrospective analysis of publicly available data with pseudonymous user information, the research team did not interact with individuals or monitor posts in real time. As a result, no crisis response protocol or intervention mechanism could be implemented if a post reflected acute suicidal distress. This limitation is inherent to retrospective analyses of publicly archived online data.

Our research team comprises researchers holding diverse gender, racial, and cultural backgrounds, including people of color and immigrants, and holds interdisciplinary research expertise. Our research team comprises computer scientists with expertise in human-computer interaction, social computing, and digital mental health, and psychologists with expertise in clinical psychology, adolescent depression and suicide, and digital health interventions. One of our psychologist coauthors specializes in suicide etiology, suicide prevention, and crisis intervention, and the other psychologist coauthor is a clinical psychologist with more than 16 years of experience spanning adult and adolescent inpatient care and crisis suicide helplines. To ensure validity and prevent misrepresentation, our findings were reviewed and corroborated by our psychologist coauthors. However, our work is not intended to replace the clinical evaluation of an individual undergoing suicidal thoughts and should not be taken out of context to conduct mental health assessments.

## Results

### Aim 1: Theory-Driven Characterization of SI

#### Classifying Posts Into IPTS Types

Our IPTS classifiers achieved strong performance (87%‐95% accuracy) where expert validation showed high agreement (88%‐94%), supporting reliability. Multimedia Appendix 1, Section S9, describes our approach of evaluating and validating the IPTS dimensions classifications in detail.

##### Distribution of SI Disclosures

Using our computational approach discussed earlier, we labeled our entire dataset of approximately 59,000 posts with SI dimensions and risk factors. Table 3 presents the distribution of these labels within our dataset. The most frequently expressed dimension was loneliness, accounting for 20.29% of posts (12,091 instances), indicating its predominant role in online SI discourse. Among the 3 risk factors, thwarted belongingness appeared in 13.71% of posts (8171 instances), making it the most prevalent risk factor. This was followed by perceived burdensomeness, which was present in 5.77% of posts (3441 instances), and acquired capability for suicide, which was identified in 3.32% of posts (1980 instances).

**Table 3.** Distribution of posts across Interpersonal Theory of Suicide (IPTS) dimensions and risk factors^a^ (N=59,607).

IPTS type	Posts, n	Corpus (%)	95% CI
Risk factor: thwarted belongingness	8171	13.71	13.44‐13.99
Dimension: loneliness	12,091	20.29	19.99‐20.59
Dimension: lack of reciprocal love	11,422	19.16	18.87‐19.46
Risk factor: perceived burdensomeness	3441	5.77	5.59‐5.96
Dimension: self-hate	9760	16.37	16.07‐16.68
Dimension: liability	10,141	17.01	16.71‐17.32
Risk factor: acquired capability for suicide	1980	3.32	3.18‐3.47
Lethally suicidal^b^	1508	2.53	2.40‐2.66

a Risk factors are derived from intersecting dimensions (threshold=0.60).

b Lethally suicidal posts exhibit all 3 risk factors.

Notably, 2.53% of posts (1508 instances) exhibited all 3 risk factors, classifying them as lethally suicidal. These posts exhibited the highest risk level, as individuals expressing all 3 risk factors are considered to be at an elevated risk for suicidal behaviors, according to the IPTS. Table S7 in Multimedia Appendix 1 presents example paraphrased posts corresponding to each IPTS dimension and risk factor. These examples illustrate how SI manifests in online discussions. Additionally, post hoc qualitative explanations are provided for each classification, ensuring transparency in the interpretability of the model’s decision-making process.

##### Topic Modeling Using BERTopic

**Figure 2.**
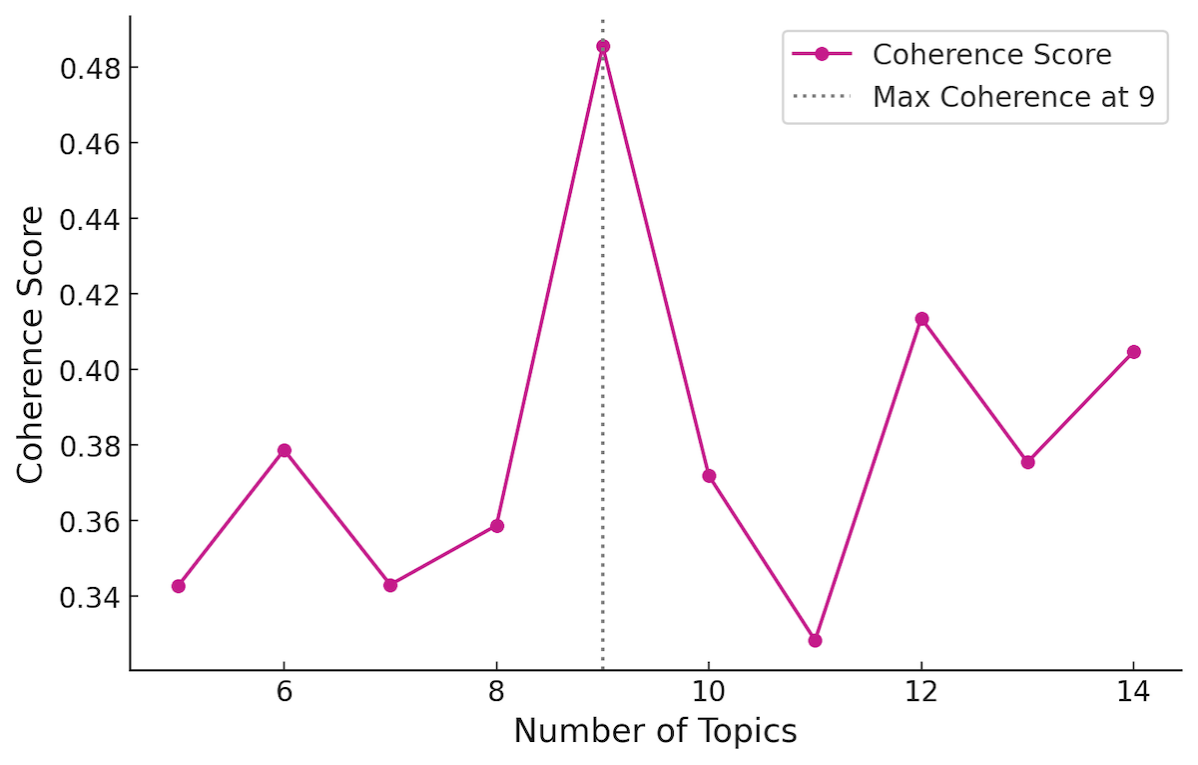
Coherence scores by varying the number of topics (**K**) in the BERTopic model.

To identify the optimal number of topics, we computed topic coherence scores for each k using the model-derived topic-word distributions and selected the k that yielded the highest coherence score (Figure 2). This optimization procedure indicated the best-performing model at k=9, which was used for all subsequent analyses reported in the *Results* section. Topic labels were assigned through manual qualitative inspection by the authors: we reviewed each topic’s most representative keywords and sample documents to generate concise topic theme names aligned with the content of each cluster. This manual labeling process was performed after the BERTopic clustering step and did not alter topic assignments.

Although coherence scores guided model selection, we did not compute CIs or bootstrapped estimates for coherence. In BERTopic, coherence is calculated after topic formation and is intrinsically tied to a single clustering solution derived from the full corpus. Generating CIs would require repeatedly resampling the dataset and refitting the entire BERTopic pipeline. As topic modeling is unsupervised and nondeterministic, each resampled run would likely produce a different topic structure, making it difficult to define a stable set of topics over which statistical uncertainty could be meaningfully estimated. As a result, such resampling would reflect variability across different models rather than uncertainty around one identified solution. Consistent with common BERTopic practice, we therefore prioritized coherence-based selection combined with qualitative interpretability checks and topic prevalence reporting and noted that more formal stability analyses remain an avenue for future work.

Consistent with BERTopic conventions, documents that could not be confidently assigned to a coherent topic were grouped into the outlier cluster Topic −1. In our final k=9 solution, the outlier cluster contained 13,061 documents, representing 9% of the topic-modeled corpus. As Topic −1 primarily consisted of noisy, sparse, or semantically heterogeneous text that could not be assigned a meaningful theme, it was excluded from thematic interpretation, resulting in 8 final labeled topics. This led to our 8 final topics, as summarized in Table 4, along with explanations and top keywords. We report the size of Topic −1 to contextualize the prevalence estimates of the remaining topics and disclose the proportion of documents not represented in the interpretable topic distributions.

**Table 4.** Topics identified as per BERTopics with explanations and top keywords.

Topic theme	Explanation	Keywords
Despair and emotional struggles	Expressions of hopelessness, dissatisfaction, and mental turmoil	don’t want life, feel like, think, anymore, lost and broken, and no hope left
Substance use	Substances such as ibuprofen or household items, potentially linked to self-harm or risky behaviors	hand_soap, drank, ibuprofen advil, advil kinda funny, mixing alcohol, and took pills again
Seeking support or validation	Highlights the need for emotional support, or recognition, suggesting a plea for interaction	need someone, need, talk, guess who back, good person, I need help, and someone please listen
Weakness and pain	Feelings of physical and emotional exhaustion, feeling broken, and recalling painful experiences	damaged weak, damaged, weak, tired, hurt, fair, worst days, and always broken
Planning and attempts	Thoughts or actions related to planning self-harm, including specific methods or failed attempts	bag tied around, tied around, xannns, failed, head, took, writing a note, and ready to jump
Nonsuicidal self-injury	Focuses on self-inflicted harm and the immediate consequences	currently bleeding everywhere, bleeding, finally, can’t stop cutting, and razor in hand
Methods and tools	Discusses methods involving specific tools or materials, possibly related to planned attempts	helium tanks, air would, oxygen mask, asphyxiation, found the rope, and sharp enough blade
Cynicism and bitterness	Expressions of cynicism and dark humor as a coping mechanism, using humor to mask pain with life	haha stay low, assholes haha, nothing ever changes, it’s all a joke, and world’s full of idiots

We obtained the distribution of these topics within our dataset and mapped the topical occurrences with risk factors and dimensions, as presented in Table S8 in Multimedia Appendix 1. The theme “general despair and emotional struggle” is prominent across all dimensions, risk factors, and lethally suicidal posts (eg, 0.0119 in lethally suicidal, 0.0145 in perceived burdensomeness, and 0.0106 in acquired capability). This suggests that individuals in emotional distress face multiple suicidal factors. It is particularly strong in the lack of love (0.0061) and loneliness (0.0017) dimensions, indicating higher emotional pain in those feeling disconnected.

Another significant theme, “seeking support or validation,” appeared across all risk factors and dimensions (eg, 0.1576 in thwarted belongingness, 0.1524 in acquired capability, and 0.1299 in lack of love). This suggests that individuals experiencing distress often reach out to OCs, possibly searching for connection, reassurance, or understanding. Its strong presence in the lack of love, loneliness, and self-hate dimensions highlights that such individuals feel deeply alienated, craving for any sort of validation to reduce their SI. This aligns with prior work that found online help-seekers often experience higher levels of psychological distress and are more likely to seek anonymity due to stigma [57].

The “feelings of weakness and pain” theme showed a strong association with lethally suicidal posts and acquired capability for suicide, despite low absolute values (eg, 0.0007 and 0.0005). This correlation highlights the link between repeated suicidal attempts and increased pain tolerance, suggesting that prolonged distress and self-harm contribute to a heightened risk of suicidal behavior. Similarly, the “nonsuicidal self-injury” theme was strongly associated with lethally suicidal and acquired capability for suicide (0.0029 and 0.0027), reinforcing the notion that individuals with a history of self-harm tend to have an increased tolerance for pain, making them more susceptible to considering or attempting suicide. This theme also appeared in the self-hate and lack of love dimensions, suggesting that negative self-perceptions and emotional deprivation contribute to self-destructive tendencies. This finding is also stated in IPTS [30].

Additionally, “cynicism and bitterness” was highly prevalent in the loneliness dimension (0.0072). This indicates that individuals who feel socially disconnected and emotionally isolated may develop a cynical worldview, potentially worsening their emotional state and reducing their willingness to seek help. This aligns with prior work that links social isolation to increased negativity and reduced engagement in support-seeking behaviors [58].

A particularly concerning theme, “planning and attempts,” was strongly associated with the risk factor of acquired capability for suicide (0.0815). This suggests that individuals who actively plan suicidal attempts often reach a critical threshold of pain tolerance. This theme indicates a transition from ideation to intent, marking a dangerous phase where individuals not only contemplate death but also take steps toward enacting it [30,42]. Overall, the presence of themes such as general despair, seeking support, and planning and attempts highlights both the urgency of intervention and the potential role of online support in shaping suicidal trajectories.

### Aim 2: Analyzing Language of Responses to SI in Online Spaces

#### Psycholinguistic Analysis of Comments (LIWC)

We analyzed the comments’ emotional tone, cognitive processes, social concerns, and other psychological factors using the LIWC-2015 lexicon [53]. Linguistic and emotional markers were identified by comparing normalized LIWC category occurrences across the 3 IPTS risk factors. Table 5 presents the distribution of these categories and the Kruskal-Wallis H-test for statistical significance. Our findings are detailed below.

The affect category reflects the emotional tone, capturing positive and negative emotions. Negative affect was highest in responses to thwarted belongingness (0.37), indicating more negative emotions while responding to those feeling socially isolated, such as in “I’m feeling in a suicidal state [...] In constant pain, and tired with zero libido, so my sex life has been wrecked too [...] Suicidal thoughts are constant here.” Responses to acquired capability for suicide exhibited a higher likelihood of sadness, as seen in a comment, “I’m very sorry all of that happened. Hope you can find hope and light in the darkness.” In this case, the responder expressed sorrow and offered sympathy to an individual who had previously attempted suicide.

**Table 5.** Normalized occurrences of psycholinguistic markers in the comments to suicidal ideation posts varying in risk factors across thwarted belongingness, perceived burdensomeness, and acquired capability for suicide, along with Kruskal-Wallis H-statistics and raw *P* values.

LIWC	Thwarted belongingness	Perceived burdensomeness	Acquired capability for suicide	H-statistic	*P* value^a^
Affect					
Positive affect	0.133	0.136	0.133	66.068	<.001
Negative affect	0.037	0.033	0.038	319.863	<.001
Anxiety	0.003	0.004	0.003	346.375	<.001
Anger	0.009	0.008	0.008	244.097	<.001
Sad	0.011	0.008	0.012	681.115	<.001
Cognition and perception					
Insight	0.034	0.029	0.031	443.637	<.001
Causation	0.017	0.017	0.019	195.374	<.001
Tentative	0.097	0.104	0.085	799.830	<.001
Certainty	0.025	0.023	0.025	338.564	<.001
Differentiation	0.099	0.097	0.095	84.117	<.001
See	0.013	0.012	0.012	661.096	<.001
Hear	0.016	0.022	0.018	165.520	<.001
Feel	0.017	0.014	0.017	281.563	<.001
Biological processes					
Body	0.012	0.011	0.014	617.260	<.001
Health	0.028	0.025	0.032	49.545	<.001
Sexual	0.008	0.008	0.006	408.176	<.001
Informal					
Informal	1.020	1.025	0.977	735.520	<.001
Swear	0.016	0.013	0.014	654.620	<.001
Assent	0.057	0.066	0.063	44.590	<.001
Nonfluent	0.073	0.083	0.068	653.310	<.001
Social and personal concerns					
Family	0.029	0.030	0.033	10.950	.004
Friends	0.011	0.014	0.011	630.290	<.001
Leisure	0.013	0.016	0.015	51.660	<.001
Home	0.004	0.003	0.004	151.970	<.001
Religion	0.004	0.004	0.005	236.080	<.001
Space	0.198	0.195	0.198	5.080	.08
Time	0.073	0.069	0.066	506.880	<.001
Achievement	0.023	0.023	0.021	567.630	<.001
Power	0.039	0.029	0.038	318.610	<.001
Function words					
Preposition	0.283	0.285	0.282	10.970	.004
Conjunction	0.147	0.142	0.141	63.010	<.001
Adverb	0.099	0.102	0.105	9.370	.009
Negation	0.037	0.033	0.044	78.900	<.001
Auxillary verb	0.195	0.197	0.199	5.390	.07
Verb	0.344	0.345	0.358	308.600	<.001
Adjective	0.105	0.099	0.109	43.150	<.001
Compare	0.048	0.050	0.054	2.830	.24
Number	0.016	0.011	0.015	477.930	<.001
Quantifier	0.044	0.048	0.049	14.190	.001
Temporal references					
Past	0.042	0.035	0.044	145.914	<.001
Present	0.302	0.311	0.316	204.571	<.001
Future	0.022	0.021	0.023	103.121	<.001

a Statistical significance is based on Bonferroni correction (adjusted α threshold=.00156).

Cognition and perception attributes include thought processes and perception-related words. Responses to perceived burdensomeness showed high usage of tentative language (0.104), indicating uncertainty, as in, “I get where you’re coming from [...] Yet because I understand doesn’t make it any less hurtful.” Responses to acquired capability for suicide also showed higher expressions of feeling (0.009), focusing on pain and suffering-related words.

Biological processes cover bodily states, health, and physical sensations. Responses to the acquired capability for suicide mentioned health the most about health (0.032), indicating physical concerns. For example, a commenter responded about their personal experience of coping with extreme sexual desires, how harming oneself is not the answer, and urged them to search for healthier alternatives. Responses to thwarted belongingness included words related to sexuality (0.008), suggesting romantic disconnection, and responses to acquired capability for suicide showed high occurrences of body-related terms (0.014), focusing on preventing self-inflicted body harm, as in, “You need to fight. Yes, I’m talking to you [...] I will never do anything to cut myself [...].”

Social and personal attributes consist of words on social connections, family, and personal interests. Responses to perceived burdensomeness focused more on friends (0.014), indicating concerns about how it affected relationships and social life, such as in, “I’ve opened up before, but it was used against me [...] Part of me misses the past relationships, but another part just wants a friend without feeling like a burden.” Responses to acquired capability for suicide mentioned religion (0.005), and power (0.038) as seen in, “During my time in the psych ward, a therapist suggested I find a religion [...] After learning about the tenets and beliefs, I decided to become a Satanist.” Also, responses to thwarted belongingness showed references to achievements (0.023) and time (0.073), reminding the individual of their accomplishments, helping them feel valued, and giving them a sense of belonging. This is seen in the example, “You’re doing your best, I can see that [...] It takes so much to get by every single day in your condition.”

Function words focus on language structure, such as articles, conjunctions, and prepositions. Responses to acquired capability for suicide are likely to use more verbs (0.358), adjectives (0.109), reflecting emotionally intense efforts by the responders to stop the individual from further harming themselves as seen in, “[...] You’re so young! [...] The only thing that’s stopping me from killing myself is the idea of another failure like, what if I failed to kill myself and end up waking in a hospital.”

Temporal references consist of time-related words, indicating thoughts’ orientation in time. For temporal references, acquired capability for suicide comments focused more on the past (0.044) and present (0.316), reflecting attention to their past, current, and future states trying to connect with the individual who posted originally. For example, one OC member narrated their personal experience, “I don’t tell people it will get better. The only thing I say is that things have gotten better for me since my last attempt [...].”

#### Content Analysis of Responses to Lethal Versus Nonlethal SI Posts

We examined content differences in responses to lethal (intersection of all 3 risk factors) versus nonlethal SI posts. To identify differences in responses, we used the SAGE [36]. SAGE compares parameters of 2 logistically parameterized multinomial models, with a self-tuned regularization parameter to balance frequent and rare terms. We applied SAGE to identify distinguishing n-grams (n=1, 2) between responses to lethal and nonlethal SI posts. The SAGE magnitude captures uniqueness-positive values indicate terms more likely in responses to lethal posts and negative values in nonlethal ones. Table 6 summarizes distinguishing keywords in responses to lethal and nonlethal SI posts. We note that the top-ranked n-grams show a relatively narrow spread of SAGE values. As SAGE quantifies lexical salience as log-frequency deviation from a background lexical distribution, conceptually distinct n-grams may receive very similar scores when they exhibit comparable levels of deviation. Thus, minor numerical differences among the highest-ranked terms should not be interpreted as substantively meaningful.

**Table 6.** Top discriminating n-grams (n=1, 2) in responses to lethally and nonlethally suicidal ideation posts with Sparse Additive Generative Model (SAGE) [36].

n-gram	SAGE^a^
life problems	5.6490
strange place	5.6462
change lot	5.6411
probiotics	5.4178
know ever	5.4177
like partner	5.4158
go pain	5.4153
grow apart	5.4144
many problems	5.4135
nothing good	5.4131
often times	5.4124
learn write	5.4122
cents	5.4121
mid-twenties	5.4117
crimes	5.4108
matter small	5.1062
penny	−5.0862
that’s okay	−4.6743
intrusive	−4.4563
intrusive thoughts	−4.4434
theyve	−4.3980
bad thing	−4.2239
views	−4.2041
find penny	−4.1867
friend like	−4.1765
caffeine	−4.1235
coworker	−4.1185
small moments	−4.1114
karma would	−4.1054
everywhere	−4.0277
go get	−4.0254
sexual life	−4.0027

a Positive SAGE indicates saliency in responses to lethally suicidal ideation posts, and negative SAGE indicates saliency in responses to nonlethally suicidal ideation posts.

We note that some extracted bigrams may appear incomplete when viewed in isolation (eg, “friend like,” “know ever,” and “go pain”). This reflects the informal, fragmented nature of user-generated social media language. Individuals expressing or responding to distress frequently write in elliptical or conversational forms (eg, “friend-like connections” and “going through pain”), and SAGE captures these recurring local word co-occurrence patterns as statistical signals. Importantly, our interpretations do not depend on any single n-gram but on converging lexical patterns across multiple related terms, which together form coherent thematic groupings that map onto meaningful psychological and interpersonal contexts.

Responses to lethal SI posts showed keywords on pain and life. Phrases such as *life problems* and *many problems* highlighted overwhelming hardships, while *grow apart* suggested isolation. *Nothing good* indicated a bleak outlook, and *body real* and *crimes* suggested victimization, likely by harming one’s body. We found a distinct pattern where commenters expressed pessimism and used depressive language, suggesting a tendency to relate the original post to personal experiences. For example, words such as *death* and *strange* pointed to exhaustion, with suicide seeming like the only escape, which could be seen in, “[..] I’m under the firm belief that life is not worth it and that death is good [..]”

In responses to nonlethal SI posts, keywords such as *intrusive thoughts* and *bad thing* reflected negative self-perceptions and feelings of being a burden. A *friend like* and *coworker* suggested superficial connections and disconnection, as seen in, “Realizing how far my avoidant tendencies go, pushing away from all my friend-like connections [..] Actively destroying my friendship over stupid stuff.” We also found occurrences of keywords such as *small moments*, which indicated assistance in helping to find joy, whereas *tell someone* indicated a desire to help individuals on the platform who have SI.

Together, this section analyzed the language used in responses to SI posts, which revealed insights into the emotional and psychological states of individuals responding. We found people respond differently to different SI posts. When the individual felt like a burden, responses were more positive but contained uncertainty. When someone felt isolated, responses were the most negative and also uncertain, reflecting doubts about unhealthy social connections. Responses to those who had attempted or thought about suicide focused more on health and power, using higher verbs and strong emotions, denoting urgent language to discourage self-harm. These responses also referenced the past, present, and future, helping build connections through personal stories. This was also observed by looking at the SAGE-based content analysis between responses to lethal and nonlethal SI posts, where responses to lethal SI posts share their own suicide attempt story.

### Aim 3: Evaluating AI’s Responses to SI

#### Comparing AI and Human-Written Responses

##### Overview

After generating AI responses across various contextual settings, we conducted a comparative analysis against human-written responses from OCs. For this purpose, we built upon previous research and performed a comprehensive suite of lexico-semantic analyses [22,59-62]. Tables7  8 present an overview of these comparisons. In addition to reporting statistical significance, we also report standardized effect sizes to quantify the magnitude of observed differences. For paired comparisons between each AI configuration and OC responses, we calculated Cohen *d* for paired samples. For comparisons across all 4 response modalities, we derived an effect size for the Kruskal-Wallis test using eta squared (η²), representing the proportion of variance attributable to group differences. Owing to the large sample size, even small differences can reach statistical significance; therefore, effect sizes are provided to aid interpretation of practical significance. All *P* values were adjusted using the Benjamini-Hochberg false discovery rate correction to account for multiple comparisons.

**Table 7.** Linguistic features: summary of comparing the responses on online communities (OC) and by multiple artificial intelligence (AI); AI-1 (default GPT-4), AI-2 (GPT-4 with themes), and AI-3 (GPT-4 with themes and characteristics of supportive responses), including *t* tests in comparison with OC responses, and a Kruskal-Wallis H-test across all the 4 modalities.

	OC, mean	AI-1, mean	AI-1 versus OC			AI-2, mean	AI-2 versus OC			AI-3, mean	AI-3 versus OC			Kruskal-Wallis H	*P* value	η²	Direction
			*t*	*P* value	Cohen *d*		*t*	*P* value	Cohen *d*		*t*	*P* value	Cohen *d*				
Verbosity (response level)	54.57	234.61	20.57	<.001	0.46	246.58	21.93	<.001	0.49	323.80	26.83	<.001	0.60	426.00	<.001	0.0529	AI>OC
Verbosity (sentence level)	14.70	17.89	3.58	<.001	0.08	18.15	3.62	<.001	0.09	18.49	4.02	<.001	0.09	177.37	<.001	0.0218	AI>OC
Readability	2.33	7.94	85.01	<.001	1.90	7.20	71.52	<.001	1.60	6.52	62.60	<.001	1.40	84.43	<.001	0.0101	AI>OC
Repeatability	0.10	0.16	4.92	<.001	0.11	0.16	5.37	<.001	0.12	0.15	4.47	<.001	0.10	50.02	<.001	0.0059	AI>OC
Complexity	9.47	15.35	5.81	<.001	0.13	15.78	6.26	<.001	0.14	16.05	6.55	<.001	0.16	112.28	<.001	0.0136	AI>OC

**Table 8.** Linguistic style and adaptability to query: summary of comparing the responses on online communities (OC) and by multiple artificial intelligence (AI); AI-1 (default GPT-4), AI-2 (GPT-4 with themes), and AI-3 (GPT-4 with themes and characteristics of supportive responses), including *t* tests in comparison with OC responses, and a Kruskal-Wallis H-test across all the 4 modalities.

	OC, mean	AI-1, mean	AI-1 versus OC			AI-2, mean	AI-2 versus OC			AI-3, mean	AI-3 versus OC			Kruskal-Wallis H	*P* value	η²	Direction
			*t*	*P* value	Cohen *d*		*t*	*P* value	Cohen *d*		*t*	*P* value	Cohen *d*				
Categorical Dynamic Index	2.52	7.22	11.22	<.001	0.50	7.31	12.3	<.001	0.55	7.87	14.5	<.001	0.65	165.43	<.001	0.060	AI >OC
Formality	0.57	0.95	7.60	<.001	0.17	0.94	7.60	<.001	0.17	0.92	7.16	<.001	0.16	78.41	<.001	0.00943	AI>OC
Empathy	0.70	0.65	−3.1	.002	−0.07	0.69	−0.9	.37	−0.02	0.70	−0.10	.92	0.00	42.22	<.001	0.0050	OC≥ AI
Semantic similarity	0.39	0.62	8.94	<.001	0.20	0.62	8.56	<.001	0.19	0.63	8.94	<.001	0.20	122.00	<.001	0.0149	AI>OC
Linguistic style accommodation	0.89	0.97	3.58	<.001	0.08	0.97	3.58	<.001	0.08	0.97	3.58	<.001	0.08	75.28	<.001	0.00901	AI>OC
Diversity	0.46	0.16	−13.42	<.001	−0.30	0.15	−13.86	<.001	−0.31	0.16	−13.85	<.001	−0.31	140.62	<.001	0.0172	OC>AI

##### Linguistic Structure

For linguistic structure, we operationalized several measures of verbosity, readability, repeatability, and complexity that we describe below.

###### Verbosity

Verbosity serves as a measure of detail and elaboration in communication, which is often associated with the effectiveness of support [22,63]. We operationalized two types of verbosity at (1) *response level* (the total number of words per response) and (2) *sentence level* (the average number of words per sentence). On average, AI responses were approximately 4 to 5 times longer and contained more words per sentence than OC (mean 54.57) responses, with statistical significance. A notable trend across the 3 AI configurations is the marked increase in verbosity with increased contextual information.

###### Readability

Readability reflects how easily a reader can comprehend a given text. In health and online health contexts, it plays a crucial role in both expression and interpretation [64,65]. We obtained the Coleman-Liau Index (*CLI*) [66] to assess readability, which evaluates character and word structure within a sentence. The *CLI* is calculated as *CLI*=(0.0588*L*−0.296*S*−15.8), where *L* represents the average number of letters per 100 words, and *S* represents the average number of sentences per 100 words. We found that AI responses showed higher readability than OC responses. Although higher readability indicates improved writing quality, it can also imply a greater educational requirement for comprehension. Interestingly, adding more contextual information led to a decrease in readability scores, with AI-3 showing the lowest readability among the AI responses. This could be indicative of the fact that adding additional context led responses to be marginally closer to human-written responses.

###### Repeatability and Complexity

Repeatability and complexity are syntactic measures that are associated with cognitive processes, such as planning, execution, and memory [64]. Repeatability refers to the frequency of word reuse, where higher values may indicate lower communication quality due to redundancy. On the other hand, complexity, measured by the average length of words per sentence, influences how effectively ideas are conveyed, with greater complexity often associated with nuanced, precise, and detailed communication [67]. Again, AI responses showed a significantly higher repeatability and complexity than OC responses. That said, both of these measures remain largely similar across AI-1, AI-2, and AI-3 responses. Importantly, because complexity was computed as a normalized structural ratio rather than a raw length-based count, the influence of overall response length on this metric is reduced.

### Linguistic Style

Next, within linguistic style, we operationalized and compared across categorical dynamic index (CDI), formality, empathy, and hopefulness in responses.

#### Categorical Dynamic Index

CDI is a bipolar linguistic measure that assesses writing style on a spectrum from categorical to dynamic [35]. We calculated the CDI of each response by obtaining the parts of speech occurrences as per LIWC-2015 [53]. A higher CDI value reflects a categorical writing style characterized by structured, abstract, and analytical expression, whereas a lower CDI signifies a dynamic or narrative style, emphasizing storytelling and fluidity. We found that AI responses exhibited approximately 200% higher CDI than OC responses. This indicates that OC members use a narrative and dynamic style of writing in responding to posts, whereas the AI uses a more categorical and analytical style of writing.

#### Formality

Formality is a key sociolinguistic feature that reflects the level of sophistication, politeness, and relevance to linguistic norms [68]. Formal language is typically structured, grammatically precise, and commonly used in professional, academic, and official settings, whereas informal language adopts a more relaxed tone, often incorporating slang, colloquialisms, and abbreviations. To assess formality, we leveraged a RoBERTa-based formality classification model [69,70] trained on Grammarly’s Yahoo Answers Formality Corpus [71], achieving a receiver operating characteristic–area under curve of 0.98 on benchmark datasets. We found that formality is exhibited with a much higher extreme in AI responses (mean >0.92) than OC responses (mean 0.57), with a statistically significant difference. Increasing contextual information for the AI resulted in similar formality scores, with a slight decrease.

#### Empathy

Empathy is a complex cognitive ability that allows individuals to understand and share the emotions and perspectives of others, playing a crucial role in supportive communication by fostering emotional connection and validation [72]. We used a RoBERTa-based model trained on empathetic reactions to news stories [73]. Interestingly, AI responses showed lower empathy than OC responses. However, with the addition of more contextual information, empathy scores get higher in AI responses, getting closer to OC responses. As empathy scores were also calculated as proportional linguistic signals rather than absolute counts, the effect of verbosity on this measure is likewise normalized and minimized.

### Adaptability to Query

Finally, we operationalized measures in how the responses adapted to the queries in terms of semantic similarity, linguistic style accommodation, and diversity.

#### Semantic Similarity

Semantic similarity measures the extent to which a response is topically and contextually similar to a post. We computed the cosine similarity between the 384-dimensional embeddings of posts and responses using a transformer-based language model, *all-MiniLM* [48]. We found that AI responses showed a significantly higher semantic similarity than OC responses. We also noted a marginal increase in semantic similarity with added context in prompting the AI.

#### Linguistic Style Accommodation

Linguistic style accommodation goes beyond content similarity and evaluates how well a response stylistically matches its query, focusing on noncontent words such as function words and pronouns [74]. Prior research showed that adapting to a user’s writing style can improve online support [21,22]. We computed the occurrences of these parts of speech using the LIWC-2015 lexicon [53]. Then, we obtained the vector representations of posts and corresponding responses on the occurrences of these parts of speech and measured the cosine similarities to quantify the linguistic style accommodation. We found that AI responses show higher linguistic style accommodation than OC responses and almost perfectly match the linguistic style of the queries (mean 0.97).

#### Diversity

Diversity refers to the uniqueness and variation in responses, and greater diversity is known to be associated with greater effectiveness in psychotherapy and social support [59,75]. To measure diversity, we computed centroid vectors from word embeddings in a 384-dimensional space using the *all-MiniLM model* [76] and measured the cosine distance of individual responses from these centroids. A greater distance indicated higher linguistic diversity, reflecting more varied and creative responses. We found AI responses show a much lower diversity than OC responses. This might be indicative of the aspect that AI tends to reuse and repurpose similar suggestions across several responses. Therefore, although AI can generate coherent and contextually relevant responses, the responses lack diversity. On the other hand, OC members are likely to provide experience-based suggestions and personal narratives, exhibiting higher diversity.

### Expert Evaluation of AI Responses to SI: Anticipating Concerns and Harms

#### Overview

It is critical to examine AI’s potential benefits and limitations in responding to individuals experiencing SI. We qualitatively explored the nuances in the AI responses and identified whether these responses could lead to possible harm. We obtained a random sample of 200 posts (and corresponding AI responses) and had these expert-appraised by our psychologist coauthors to provide detailed assessments. This subset of 200 posts was randomly drawn from the larger 2000-post AI evaluation set to ensure representation across IPTS risk factors and prompting conditions. The purpose of this subset was in-depth clinical and theoretical appraisal rather than statistical generalization, as expert evaluation of therapeutic nuance, safety orientation, and theoretical alignment is substantially more time intensive than automated linguistic analysis.

To improve methodological transparency, the expert evaluation followed a structured coding rubric grounded in cognitive behavioral therapy, the SAFE-T suicide risk framework, the Collaborative Assessment and Management of Suicidality model, and the IPTS. Each response was reviewed across four domains: (1) emotional and cognitive support (cognitive behavioral therapy informed), including emotional validation and avoidance of reinforcing maladaptive cognitions [77]; (2) suicide safety orientation (SAFE-T informed), including recognition of suicide risk signals and appropriateness of crisis resource recommendations [78]; (3) collaborative and person-centered engagement (Collaborative Assessment and Management of Suicidality informed) [79], including personalization, respect for autonomy, and collaborative tone; and (4) theoretical risk alignment (IPTS informed), assessing whether responses appropriately addressed signals of thwarted belongingness, perceived burdensomeness, and acquired capability for suicide and whether response intensity matched implied suicide risk. Each domain was rated on a 3-point scale (0=absent or inadequate, 1=partially present, and 2=clearly present or appropriate) to support systematic comparison across AI prompting conditions.

In applying the IPTS-informed component of the rubric, psychologists specifically examined whether AI responses acknowledged and addressed the interpersonal risk signals reflected in the original post. For example, posts expressing social isolation were evaluated for efforts to foster connection and belonging, posts reflecting self-hate or liability were assessed for language that reduced self-blame and perceived burdensomeness, and posts indicating acquired capability for suicide or lethally suicidal intent were examined for appropriate safety orientation and encouragement of external support. Using IPTS as an evaluative lens ensured that expert judgments were theoretically aligned with the same suicide risk framework used throughout the study.

Psychologist evaluators first reviewed responses independently and then discussed discrepancies to reach consensus. As consensus coding was used rather than fully independent dual coding, interrater reliability statistics are not reported, and this is acknowledged as a limitation. We also note that future work should incorporate blinded, independent raters, and formal reliability assessments to further strengthen methodological rigor. On the basis of their comments, we grouped the observations into the following key themes.

#### Emotional Alignment and Response Effectiveness

AI-3, which was prompted with the SI post, SI category, and linguistic characteristics of supportive responses, exhibited responses with stronger emotional alignment with user distress compared to AI-1 and AI-2. It used more explicitly empathetic language, such as “I truly feel for you” or “I can imagine how difficult this must be.” These linguistic markers suggest an effort to build rapport and validate emotions, which can be crucial in fostering trust in digital interventions. However, while AI-3’s responses were perceived as more compassionate, the improvements over AI-1 and AI-2 were often subtle rather than substantial. Despite its increased emotional alignment, AI-3, like its counterparts, sometimes defaulted to generalized supportive statements such as “I’m sorry to hear that,” which could feel impersonal. In a small proportion of cases, AI-generated responses contained no additional text beyond a few such broadly supportive statements. The chatbot’s tendency to rely on preformulated expressions limited its ability to engage meaningfully in nuanced conversations, highlighting a fundamental challenge in AI-mediated crisis support, balancing emotional alignment of responses with conversational depth. Recent work has shown that LLMs often simulate empathy in exaggerated or overly formal ways that feel performative rather than genuine [80-84]. These findings align with the subtle overuse of empathetic markers observed in AI-3, suggesting that the challenge lies not in generating empathy, but in calibrating it to the context and emotional needs of users experiencing SI.

#### Personalization and Trust-Building

A recurring concern across all AI models was the lack of personalization in responses. While none of the AI-generated replies were overtly harmful, many lacked specificity in addressing the unique concerns of each user. This was particularly evident when AI-generated responses failed to acknowledge key details in posts, such as prior negative experiences with mental health professionals or distrust of medical systems. For instance, when users expressed disillusionment with therapy, the chatbot frequently recommended seeking professional help without adapting its response to account for the user’s reservations. Prior studies emphasize that genuine empathy and trust in AI-mediated conversations arise from personalization and context-sensitive adaptation [82,85]. When LLMs rely on generic reassurance rather than reflective listening, users may perceive them as emotionally distant or dismissive. Addressing this limitation requires not only linguistic variation but an awareness of users’ prior disclosures and situational context to sustain authentic and safe engagement.

#### Shifting Between Supportive Listening and Intervention

One notable finding was the variation in how AI models determined when to shift from empathetic engagement to recommending crisis resources. Posts containing explicit references to prior suicide attempts or methods did not always elicit a shift in AI responses toward immediate intervention. While AI-2 and AI-3 were more likely to encourage users to trust hospital-based treatment providers, AI-1 occasionally prompted further conversation without recommending professional support. This inconsistency raises ethical considerations regarding AI-driven risk assessment. Future research should explore optimal strategies for balancing empathetic engagement with timely intervention while avoiding responses that feel formulaic or dismissive.

#### Validation Without Reinforcing Harmful Cognitions

An encouraging finding was that none of the AI models explicitly reinforced SI or validated harmful cognitive distortions. However, subtle differences emerged in how the models addressed suicidal thoughts. For instance, in response to posts asserting that suicidal thinking is “normal,” AI-2 explicitly countered this notion by stating, “Feeling suicidal is *not normal*, and wanting to harm yourself isn’t something you should cope with in silence.” In contrast, AI-3 was less direct in challenging such assertions. While pushing back against harmful beliefs can be beneficial, the way this is done matters. Responses that feel overly clinical or detached, such as AI-2’s phrasing in some cases, may risk alienating users who seek emotional validation. Future iterations of AI-driven crisis support should focus on balancing between validating distress and gently guiding users toward reframing harmful thoughts in a nonconfrontational manner.

#### Variability Across AI Models

Although AI-3 displayed a slight tendency toward more expressive empathy, the overall differences between the 3 models were not always stark. The variation in responses was often more attributable to the nature of the user’s post rather than fundamental differences in AI architecture. Given this variability, further analysis is required to determine whether specific fine-tuning strategies consistently enhance AI-driven support systems.

#### Potentially Less Effective Responses

Despite their generally supportive nature, some AI responses were less helpful due to issues in phrasing or tone. Responses such as “Our minds can sometimes trick us into believing things that aren’t true, especially when we’re feeling down” (AI-1) risked sounding dismissive rather than reassuring. Similarly, AI-generated responses about the user in the third person (eg, “You mentioned that you aren’t scared after your attempt. This can be deeply concerning, as it might indicate an increased risk of attempting again.”) could feel impersonal and detached.

#### Balancing Response Length and Engagement

A final consideration is the optimal length and depth of AI-generated responses. While generic responses were sometimes perceived as less helpful, overly lengthy replies also risked being impractical. Users who posted brief messages often received disproportionate length responses, potentially making engagement feel unnatural. A more conversational approach—where response length aligns with the user’s post and includes follow-up questions—may enhance interaction quality while preserving the chatbot’s role as a supportive entity rather than an information dispenser.

Overall, although none of the responses in our evaluated sample contained overtly harmful or explicitly unsafe instructions, LLMs are known to occasionally produce inaccurate, overgeneralized, or non–evidence-based statements, particularly in complex mental health contexts. In suicide-related conversations, such inaccuracies could involve inappropriate reassurance, incomplete framing of treatment options, or the omission or incorrect presentation of crisis resources. The possibility of AI “hallucinations,” where responses sound plausible but are not clinically grounded, is especially concerning in high-risk situations where users may rely on the information provided. Furthermore, the substantially greater verbosity of AI responses, often 4 to 5 times longer than human replies, has implications for usability. Individuals in acute distress may experience reduced attention, cognitive overload, or emotional fatigue, making lengthy responses harder to process despite their structural coherence. These considerations highlight that, beyond empathy and tone, AI systems intended for suicide-related support must be evaluated for informational reliability, crisis safety alignment, and cognitive accessibility.

## Discussion

### Principal Findings

This study offers a comprehensive, theory-driven investigation into SI as expressed and responded to in OCs, combining computational modeling, psycholinguistic analysis, and comparative evaluation of human and AI-generated responses. Guided by the IPTS, our findings highlight the psychological, social, and technological dynamics that shape suicidal expression and support in digital environments. Through this lens, we extend the applicability of established suicide theories to online contexts, uncovering both the continuities and the distinctive nuances of digital mental health discourse.

Our analyses demonstrated strong empirical support for the applicability of the IPTS framework in large-scale online data. Posts labeled under perceived burdensomeness, thwarted belongingness, and acquired capability for suicide displayed linguistic and cognitive patterns consistent with those theorized by IPTS, underscoring its relevance for understanding online expressions of suicide risk. A high correspondence between manual and computational classifications reaffirmed the model’s theoretical coherence in this context. Posts associated with lethal intent or high-risk behaviors frequently contained explicit references to pain, weakness, self-harm, and methods or planning, reflecting the acquired capability for suicide construct. This prominence of planning- and attempt-related language in high-risk posts may be understood in light of how acquired capability for suicide manifests behaviorally, as individuals with greater exposure to pain and prior self-harm may be more likely to articulate concrete thoughts about methods or preparation. These findings align with prior evidence suggesting that suicide attempters exhibit greater pain tolerance and a history of exposure to self-harm than ideators alone [79,86,87]. Beyond IPTS, the observed progression from emotional distress to behavioral planning parallels the 3ST [88] and the IMV model [89], suggesting that online disclosures follow the same motivational and volitional trajectories of risk previously observed in clinical and offline settings.

Linguistically, suicidal disclosures online were found to be multifaceted and deeply affective. The dominant thematic cluster, general despair and emotional struggle, appeared across dimensions, reflecting the central role of psychological pain in SI. Other clusters, such as seeking support or validation and feelings of weakness and pain, illustrated both vulnerability and an underlying effort to reestablish social connection. Themes tied to planning and attempts and nonsuicidal self-injury represented the threshold between ideation and enactment, capturing the subtle but critical transitions in suicidal cognition. Importantly, these themes reveal that individuals in online spaces do not merely express distress; they also actively negotiate meaning, belonging, and self-worth within a communal framework.

Our analysis of peer responses to suicidal posts (aim 2) revealed that OCs, despite their informality, often enact spontaneous yet structured forms of social support. Linguistic patterns indicated that responses to perceived burdensomeness tended to be more positive and reassuring, while those to thwarted belongingness mirrored the poster’s distress, possibly as an empathic alignment mechanism. Phrases such as “that’s okay,” “you’re not alone,” and “it can change” were common across responses, suggesting an orientation toward emotional validation and encouragement. Responses to acquired capability for suicide posts often included religious, existential, or achievement-related themes, reflecting attempts to redirect attention toward meaning-making and resilience. These findings illuminate that online empathy, while widespread, can also exhibit limits; responses sometimes generalized or understated the severity of distress, revealing a tension between care intent and emotional attunement. Nonetheless, these peer interactions align with therapeutic communication strategies known to mitigate acute distress [90-92], highlighting the emergent therapeutic potential of peer-based online spaces.

Comparatively, the evaluation of AI-generated responses (aim 3) revealed that while current LLMs demonstrate strong structural and linguistic competence, they continue to fall short in emotional authenticity and contextual sensitivity. AI responses were coherent, grammatically polished, and semantically aligned with user posts; yet, they lacked the nuanced warmth and personalized empathy characteristic of human responses. Their tone tended to be formal, repetitive, and generalized, attributes that, although safe and well-intentioned, may render them emotionally distant in crisis contexts. Psychologist evaluations confirmed that these limitations often stem from the AI’s absence of lived experience and an inability to fully simulate human emotional reciprocity. Nevertheless, models that incorporated contextual and linguistic cues displayed modest improvements, offering responses that were more emotionally aligned and less mechanical. These findings suggest that while AI chatbots can serve as preliminary support tools, providing structured guidance, de-escalation, or triage in the absence of immediate human help, they must evolve toward greater emotional adaptivity and transparency to be effective in sensitive mental health interactions.

Beyond emotional limitations, important considerations also relate to the factual reliability and safety of AI-generated support in crisis contexts. Although the AI responses analyzed in this study were generally supportive in tone, LLMs remain prone to producing inaccurate or non–evidence-based statements, a phenomenon often referred to as hallucination. In high-risk suicide contexts, such inaccuracies could include providing incorrect crisis resources, oversimplified therapeutic advice, or statements that appear clinically authoritative without appropriate grounding. Even when well intentioned, such responses may inadvertently mislead vulnerable individuals or delay access to professional care. Another practical concern is the substantially greater verbosity of AI responses, which were, on average, 4 to 5 times longer than human replies. While longer responses may appear more informative, they can also increase cognitive load for individuals in acute distress, who may struggle to process dense or extended text. This raises important design questions regarding optimal response length, clarity, and prioritization of actionable, safety-oriented information. Together, these considerations highlight that AI systems in suicide prevention must be evaluated not only for empathy and tone but also for accuracy, safety, and usability under conditions of emotional crisis.

The broader theoretical implications of our findings extend suicide research into digital domains. While traditional frameworks conceptualize SI primarily in offline contexts, our results illustrate that the online environment transforms these experiences through anonymity, persistence, and community validation. Digital affordances enable individuals to express distress more openly, receive asynchronous empathy, and construct reflective narratives about their struggles, experiences that can both alleviate and reinforce feelings of isolation. The online environment, thus, reshapes how belongingness and burdensomeness are experienced and negotiated, suggesting that IPTS and related models can be refined to capture the sociodigital dimensions of suicidal expression.

At a practical level, our results underscore the potential of theory-informed design for online mental health platforms. Embedding IPTS constructs into community design could allow for adaptive moderation tools that detect high-risk expressions of perceived burdensomeness or belongingness loss, prompting timely peer or professional intervention. Training peer supporters to identify linguistic markers associated with specific IPTS dimensions could further enhance community responsiveness and inclusivity. In parallel, AI systems designed for crisis contexts should balance computational precision with emotional resonance, incorporating context memory, adaptive phrasing, and transparent disclosure of their nonhuman nature to foster ethical trust. AI models can also serve as simulation tools for training mental health volunteers, providing realistic conversational practice grounded in the linguistic patterns identified in this study.

In addition, IPTS-informed computational signals could be operationalized within platform triage pipelines as decision support tools for human moderators rather than autonomous diagnostic systems. For example, posts exhibiting multiple co-occurring IPTS risk factors (eg, perceived burdensomeness combined with thwarted belongingness) or language associated with acquired capability for suicide could be algorithmically prioritized for rapid human moderator review. Posts containing markers of imminent risk, such as explicit planning or method-related language, could trigger immediate escalation workflows, including the prominent display of crisis resources or alerts to trained crisis responders embedded within moderation teams. In this model, AI functions as an early-warning and prioritization layer that improves response timeliness while preserving human oversight in high-stakes decisions.

Such IPTS-based scoring mechanisms could also support tiered intervention pathways. Lower-risk posts might be routed toward peer-support engagement or automated check-in prompts, whereas higher-risk posts could prompt proactive outreach from trained volunteers or clinicians where such infrastructure exists. Importantly, these systems should be explicitly framed as screening and prioritization aids rather than diagnostic instruments, operating within clearly defined human-in-the-loop governance structures that ensure final judgment rests with trained personnel.

Regulatory and liability considerations further shape the responsible deployment of these technologies. When AI systems influence the prioritization of mental health–related interventions, they may intersect with emerging regulatory frameworks governing clinical decision support and digital health tools, depending on deployment context and functional claims. Developers and platform operators should therefore document model limitations, maintain audit trails, conduct ongoing performance and bias monitoring, and clearly delineate that AI outputs are advisory rather than determinative. Establishing transparent escalation protocols and clarifying the supportive (not clinical) role of AI can help mitigate legal and ethical risks while aligning these systems with best practices in digital mental health governance.

Ethically, our findings reaffirm that the use of AI in suicide prevention must proceed with caution. Risks of misclassification, bias, and hallucination carry profound implications for individuals in distress. Models trained on general-purpose datasets may misinterpret cultural or linguistic nuances, leading to inappropriate or harmful responses. Moreover, the potential misuse of data for nonclinical purposes, such as advertising or insurance profiling, underscores the importance of privacy, informed consent, and compliance with data protection regulations such as General Data Protection Regulation and Health Insurance Portability and Accountability Act. Transparency about the role of AI, data handling, and the limits of automated empathy is essential for maintaining user trust and preventing harm in vulnerable populations.

Overall, this study establishes that SI online embodies both enduring theoretical principles and emerging digital dynamics. While the fundamental constructs of IPTS, perceived burdensomeness, thwarted belongingness, and acquired capability for suicide remain robust explanatory anchors, their manifestation in online discourse reflects unique psychosocial and technological influences. Human responders exemplify relational empathy that AI systems have yet to replicate, but both play complementary roles in a digitally mediated ecosystem of support. By bridging theoretical psychology, computational linguistics, and AI ethics, this work contributes an integrative understanding of how SI is expressed, recognized, and potentially supported in an increasingly digital world.

### Comparison With Prior Work

In prior work on SI, foundational ideation-to-action frameworks such as the IPTS [30], the 3ST [89], and the IMV model [89] explain how SI progresses into behavior, emphasizing factors such as thwarted belongingness, perceived burdensomeness, psychological pain, hopelessness, and acquired capability [30,88,89]. Despite their explanatory value, most empirical validations rely on clinical interviews or self-reported instruments such as the Columbia-Suicide Severity Rating Scale [86] and the Patient Health Questionnaire [93], which are limited by stigma, privacy concerns, and sociocultural variation, leading to underreporting and reduced scalability [94-97]. To address this, our study operationalizes IPTS constructs using natural language analyses of online self-disclosures.

Building on prior research on the internet’s role in enabling self-disclosure and social connectedness [8,12,22,98-101], we situate SI expression within online peer-support communities. Social support, as defined by the Social Support Behavioral Code, includes emotional, informational, esteem, network, and tangible forms [20-23,102-107], with emotional and informational support most prevalent. Consistent with psycholinguistic and therapeutic work on empathy, warmth, and therapeutic alliance [35,59,108-112], prior studies show that online interactions mirror traditional counseling patterns [21,59,113]. Our findings show that empathy, immediacy, and emotionality characterize responses to SI disclosures, while linguistic markers of distress predict offline outcomes such as hospital visits and counseling utilization [75,114-119].

Prior computational studies have also explored SI detection on social media using methods from psycholinguistic features to neural models [120-124]. For example, LIWC-based analyses [125] and deep neural networks [126] identify suicidal themes and psycholinguistic differences [40,127]. Building on this, our study uses a theory-driven TopicBERT approach to cluster SI discourse and align themes with IPTS dimensions, complementing work integrating psychological theory with computational methods [54-56]. By combining ideation-to-action frameworks with topic modeling, we capture cognitive-affective signals of SI alongside patterns of peer support, bridging theory and real-world digital behavior.

This work advances prior studies on suicide risk detection and psychological modeling in social media data [128-130], moving beyond their demonstration of inferring psychological risk signals from text through key methodological, theoretical, and ethical innovations. Earlier datasets were drawn from historical Reddit archives, often predating 2016, and limited to decontextualized post-level analyses [128,129]. In contrast, this work constructs a dataset of 59,607 posts and 149,144 comments from r/SuicideWatch between May 2023 and February 2024, preserving thread structure, timestamps, and metadata to enable discourse-level modeling of SI and social interaction. Our work is also motivated by Shing et al, which introduced theory-inspired features by linking linguistic entropy to IPTS constructs [130]. Methodologically, our framework goes beyond binary SI detection: (1) interpretable IPTS-based modeling of ideation, (2) linguistic characterization of community responses using LIWC and SAGE, and (3) evaluation of LLMs such as GPT-4o in responding to at-risk disclosures. This final component bridges social media research and AI ethics by systematically assessing generative model responses to suicidal content using theory-grounded cues, a dimension not previously explored in computational suicidology.

Parallel to these contributions, growing work examines technology-mediated mental health interventions. Computer-assisted psychotherapy and social media approaches improve well-being through linguistic strategies emphasizing adaptability, creativity, and empathy [22,64,113,131,132]. Our work builds on this by contrasting linguistic mechanisms of human support with those generated by AI, directly comparing organic empathy and algorithmic responses to SI disclosures.

Recent interest in AI-driven conversational agents, driven by shortages of clinical resources [31-34,133-139], highlights their potential for scalable, immediate support using transformers and sentiment analysis [134-136]. For example, Replika, based on GPT-3 and GPT-4, shows benefits in well-being and suicide prevention contexts [137]. However, challenges remain in reliability, contextual understanding, and clinical integration [133,138,139], with studies noting risks of superficial empathy or inappropriate reassurance [13,140-142]. Although LLMs can emulate psychiatric questioning, they are limited in diagnosis and risk stratification [143], contributing to clinician concerns around accuracy, ethics, and safety [144-146]. Accordingly, our study evaluates strengths and limitations of LLM responses to SI disclosures, identifying gaps between algorithmic empathy and human compassion, and providing expert-validated insights for responsible AI design in suicide prevention.

Taken together, this work extends prior literature across three domains: (1) computationally operationalizing established suicide theories (IPTS, 3ST, and IMV) within naturalistic online discourse; (2) examining linguistic markers of emotional and informational support aligned with the Social Support Behavioral Code framework; and (3) empirically evaluating AI-driven interactions alongside human responses to suicidal disclosures. Our study integrates theoretical, linguistic, and technological dimensions to provide a holistic understanding of how SI manifests and is addressed in online spaces. This synthesis advances the field toward theory-informed, ethically aligned, and data-driven models of mental health support that bridge identification, empathy, and intervention in the age of AI.

### Limitations

Our study has limitations, which also suggest interesting future directions. In the case of SI, our study is limited by what can be observed from online data alone. First, despite corroborating our findings with psychologists specializing in SI, the lack of complementary information, such as clinical assessments or physiological data, prevents us from making clinical claims about SI. Our data lack formal clinical validation based on established diagnostic frameworks, such as the DSM-5 [147] or RDoC [148]. While our findings offer valuable insights, we caution against drawing direct clinical or diagnostic inferences. Nonetheless, this work can serve as a foundation for future research, including replication studies in clinical settings.

Second, our work does not empirically assess the effectiveness of AI-generated responses on individuals experiencing SI. As this remains a nascent field with many unknowns, our study used retrospectively collected data, combining quantitative analyses with expert-led qualitative assessments of relevance and supportiveness in the language of responses. Our work inspires future research in understanding the effectiveness of AI in SI intervention through deployment and experimental studies (with sufficient human supervision) in terms of how these technologies can impact the well-being of individuals. Future research can incorporate direct feedback from various stakeholders, including mental health professionals, individuals experiencing SI, moderators, and platform owners, in designing effective online support interventions. Future work should also compare AI-generated responses with curated or expert-identified high-quality human responses, enabling evaluation not only against typical peer support but also against best-practice benchmarks for supportive communication in SI contexts.

Third, our study likely has self-selection bias, as it includes only individuals who chose to disclose SI within OCs and may therefore not capture the full spectrum of individuals experiencing SI who could benefit from digital mental health interventions. Additionally, because linguistic features of suicidal disclosures and the responses they receive may vary across platforms beyond r/SuicideWatch, future research should examine more diverse contexts, including private messaging environments (eg, WhatsApp) and online therapy settings. Furthermore, expert evaluations of AI responses and classification validity were conducted by coauthors who were aware of the study design and model outputs; the absence of blinded independent raters may introduce confirmation bias. Future work should incorporate external evaluators and blinded assessment procedures to strengthen objectivity and generalizability.

Fourth, our dataset excludes posts that were deleted by users or removed by moderators prior to data collection. In highly moderated communities such as r/SuicideWatch, posts expressing imminent intent, explicit methods, or acute crisis situations may be removed for safety and policy reasons. As a result, the “lethally suicidal” category analyzed in this study may reflect a moderated or platform-surviving subset of high-risk ideation rather than the full spectrum of acute, real-time suicidal intent. This survivorship bias may lead to an underrepresentation of the most severe cases and should be considered when interpreting the prevalence and linguistic characteristics of high-risk posts. Future research in collaboration with platform moderators or crisis services, where ethically and legally appropriate, could help provide a more complete picture of acute suicidal expression online.

Fifth, our analysis was restricted to English-language posts, and all computational modeling and linguistic measures were derived from monolingual English text. As a result, our findings may not generalize to multilingual or non-English-speaking communities, where expressions of SI may differ linguistically, culturally, and contextually. Prior research suggests that Reddit mental health communities, including r/SuicideWatch, are disproportionately composed of users from Western, English-speaking countries and tend to skew toward younger age groups and male users, although precise demographic characteristics cannot be verified due to platform anonymity [101]. These platform-level demographic tendencies may influence both how distress is expressed and how support is offered, potentially limiting the representativeness of the observed patterns.

Sixth, the study relied on data collected over a 9-month window, which may reflect temporal influences specific to that period. Although no single external event defined the dataset, broader social, cultural, or platform-level changes during that time could have shaped patterns of online disclosure and response. Future longitudinal studies spanning multiple periods could help determine the stability of the linguistic and theoretical patterns identified here.

Finally, our evaluation reflects the capabilities of GPT-4o at the time of analysis (late 2024); newer models with enhanced reasoning, safety alignment, or dialogue optimization may perform differently in crisis-support contexts. Therefore, these findings should be interpreted as time bounded, and ongoing reassessment of AI systems is necessary as model capabilities continue to advance.

### Conclusions

This study examined SI in OCs through a theory-driven and computational lens. For aim 1, we showed that expressions of SI in online self-disclosures reflect meaningful psychological patterns consistent with the IPTS. Linguistic indicators of perceived burdensomeness, thwarted belongingness, and acquired capability for suicide were identifiable at scale, and high-risk posts exhibited language suggestive of behavioral capability for self-harm. At the same time, broader themes of despair, social disconnection, and diminished self-worth emerged across risk categories, underscoring the complex and layered nature of online suicidal expression.

For aim 2, our findings revealed that responses to SI disclosures in online spaces frequently conveyed empathy and emotional support, but varied systematically depending on the type of distress expressed. Responses to posts reflecting thwarted belongingness tended to mirror distress and uncertainty, whereas responses to perceived burdensomeness often emphasized reassurance, and those associated with acquired capability for suicide displayed greater urgency and personal disclosure. These patterns highlight both the strengths and limitations of informal peer support in digital mental health contexts.

For aim 3, we found that AI-generated responses demonstrated strong structural and linguistic competence, producing coherent and contextually aligned messages. However, compared to human responses, AI outputs were more verbose, repetitive, and emotionally generalized, lacking the nuanced warmth and personalization characteristic of human empathy. These results suggest that while AI chatbots may offer scalable, preliminary support, they should complement rather than replace human responders, particularly in high-risk situations.

Overall, this work illustrates how theory-informed computational methods can deepen our understanding of suicidal expression and support in online environments, and it emphasizes the need for ethically grounded integration of AI tools within broader, human-centered digital mental health ecosystems.

## Supplementary material

10.2196/86265 Multimedia Appendix 1 Additional methodological details.

## Abbreviations

AI: artificial intelligence
API: application programming interface
CDI: Categorical Dynamic Index
CLI: Coleman-Liau Index
IMV: integrated motivational-volitional
IPTS: Interpersonal Theory of Suicide
LIWC: linguistic inquiry and word count
LLM: large language model
OC: online community
RAKE: Rapid Automatic Keyword Extraction
SAGE: Sparse Additive Generative Model
SI: suicidal ideation
