# Supplementary material for: Suicidal Ideation in Online Spaces Through the Lens of Interpersonal Theory of Suicide: Exploratory Study of Self-Disclosure, Peer Support, and AI Responses
Source: JMIR AI. 2026 Jun 3;5:e86265. doi: 10.2196/86265 (PMC13232919; doi:10.2196/86265)
Supplement: Multimedia Appendix 1 [file ai-v5-e86265-s001.docx]

## Supplementary Materials

Table S1. Full system and user prompts used to generate AI responses under the three experimental prompting conditions, illustrating how contextual psychological information and supportive communication guidance were progressively incorporated.

| **AI Condition** | **Full Prompt Provided to the AI Model** |
| --- | --- |
| AI-1 (Post Only) | System message: “You are a supportive peer responding to someone who has shared thoughts related to suicide or severe emotional distress. Your role is to provide the best response.”  User message: [Reddit post text describing suicidal ideation] |
| AI-2 (Post + IPTS Category) | System message: “You are a supportive peer responding to someone who has shared thoughts related to suicide or severe emotional distress. The psychological theme reflected in the post is: [IPTS category]. This theme is commonly associated with the following emotional and cognitive experiences: [brief IPTS category description]. Provide the best response.”  User message: [Reddit post text describing suicidal ideation] |
| AI-3 (Post + IPTS Category + Supportive Communication Guidance) | System message: “You are a supportive peer responding to someone who has shared thoughts related to suicide or severe emotional distress. The psychological theme reflected in the post is: [IPTS category]. This theme is commonly associated with the following emotional and cognitive experiences: [brief IPTS category description]. Effective supportive responses in these contexts are empathetic, emotionally validating, hopeful without being dismissive, and linguistically accommodating to the person’s situation. Craft a response that reflects warmth, understanding, and supportive presence while avoiding clinical directives or authoritative advice.”  User message: [Reddit post text describing suicidal ideation] |

Table S2. Number of posts classified into each IPTS category at different similarity thresholds, calculated by applying the percentage changes in Table 2 to the baseline counts.

| **Threshold** | **Thwarted Belongingness** | **Perceived Burdensomeness** | **Acquired Capability for Suicide** | **Lethally Suicidal** |
| --- | --- | --- | --- | --- |
| 0.55 | 8,825 | 3,820 | 2,277 | 1,840 |
| 0.60 (Baseline) | 8,171 | 3,441 | 1,980 | 1,508 |
| 0.65 | 7,599 | 3,097 | 1,723 | 1,221 |

Table S3. Proportion of overlapping posts assigned to the same IPTS category when comparing classifications generated using the baseline threshold (0.60) and alternative thresholds.

| **Threshold Pair** | **Overlap** |
| --- | --- |
| 0.60 vs 0.55 | 0.78 |
| 0.60 vs 0.65 | 0.72 |

Table S4. Final size of the iteratively expanded IPTS codebooks after RAKE-based keyword extraction and expert review. Values indicate the total number of unique keywords and key phrases retained for each IPTS Dimension and RiskFactor following convergence of the codebook refinement process.

| **Dimension** | **Codebook Size** |
| --- | --- |
| Lack of Reciprocal Love | 725 |
| Loneliness | 741 |
| Self-Hate | 750 |
| Liability | 728 |
| Acquired Capability for Suicide | 630 |

Figure S5. Overview of the iterative codebook development and labeling pipeline. The process begins with an initial theory-informed codebook, followed by semantic similarity matching between posts and codebook terms to assign provisional dimension labels. Candidate keywords are then extracted and reviewed by experts to refine the codebook. This loop continues until the codebook stabilizes. The finalized codebook is subsequently used to compute dimension scores and identify suicide risk factors in posts.


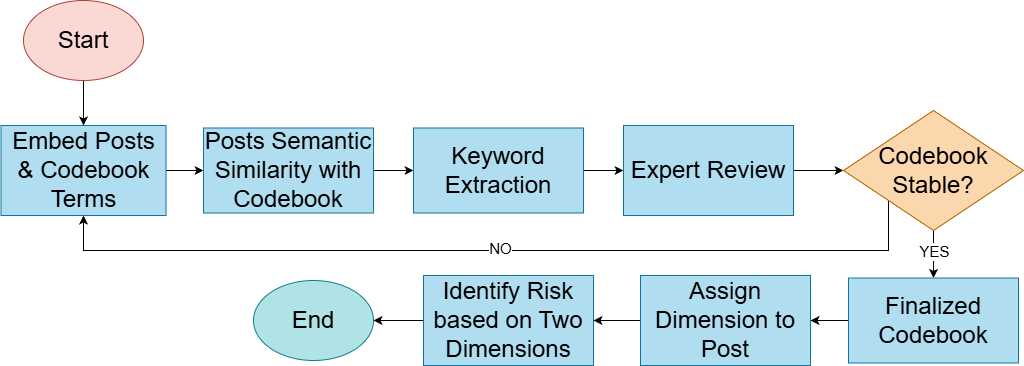


Table S6. Confusion matrix counts from expert validation of automated labels against manual psychologist annotations, showing agreement patterns across four IPTS Dimensions and four suicide RiskFactor categories (50 posts evaluated per label).

| **Category** | **TP** | **FP** | **FN** | **TN** | **Agreement** |
| --- | --- | --- | --- | --- | --- |
| **Loneliness** | 23 | 2 | 1 | 24 | 94% |
| **Lack of Reciprocal Love** | 24 | 1 | 2 | 23 | 94% |
| **Self-Hate** | 22 | 2 | 1 | 25 | 94% |
| **Liability** | 20 | 3 | 3 | 24 | 88% |
| **Thwarted Belongingness** | 21 | 3 | 3 | 23 | 88% |
| **Perceived Burdensomeness** | 21 | 3 | 3 | 23 | 88% |
| **Acquired Capability for suicide** | 18 | 5 | 5 | 22 | 80% |
| **Lethally Suicidal** | 16 | 8 | 5 | 21 | 74% |

Table S7. Examples of (paraphrased) posts for each Dimension and RiskFactor as per IPTS, along with explanations. The phrases in bold are highlighted to show verbatim phrases in the actual example post for transparency.

| **Dimension / Risk Factor** | **Example Post** | **Explanation** |
| --- | --- | --- |
| **Dimension: Loneliness** | *I'm lost and feel empty* ***without purpose or joy****. I used to have a partner who made life meaningful, but now everything feels* ***pointless****. [..] Maybe* ***she left*** *because I wasn't going anywhere. Even though no one reads this, I still* ***write my thoughts*** *because I'm too* ***afraid to end it all****. I don't think I have it in me to change.* | This post reveals deep feelings of loneliness, where the author is isolated and overwhelmed by emotional pain following the loss of a partner. |
| **Dimension: Lack of Reciprocal Love** | *I have a* ***good life*** *but struggle with* ***low self-esteem*** *and can't understand* ***how anyone, including my brothers, could love me****. [..] Despite putting my* ***pain into art****, it’s not enough, and I often visualize harming myself. [..] I want to live, but not like this, and I* ***can't afford therapy*** *or ask my* ***mother*** *for help again.* | The individual craves affection and validation but feels unworthy of love. This emotional disconnect deepens feelings of isolation, reinforcing a belief that love is conditional or unattainable. |
| **Dimension: Self-Hate** | *I'm giving it until* ***February*** *to decide if I should stay. After years of struggling with* ***mental health****, [..] I* ***despise myself, my body, and my personality****. [..] After a* ***sexual assault in March****, I feel like everything is my fault. Despite seeking help,* ***nothing worked, and I no longer want to improve****.* | Reflects deep self-hatred stemming from mental health struggles and trauma. Feelings of worthlessness, guilt, and failed attempts to improve lead to a belief that they are a burden and hold no value. |
| **Dimension: Liability** | ***I’m 17 and all I want is to die****. [...] My health is a* ***mess****—I* ***don’t exercise, my sleep is terrible, and I’m always tired****. I have to rely on my* ***parents for money****, which makes me feel* ***guilty****. I know something’s* ***wrong with me****, but I have* ***no idea*** *how to fix it.* | Conveys a belief that achievements are worthless due to financial struggles, health issues, or personal dissatisfaction. The author feels unable to meet societal expectations, leading to guilt and a sense of letting others down. |
| **Risk Factor: Acquired Capability for Suicide** | *I’ve made up my mind about* ***killing myself****. [...] I’ve been* ***cutting myself*** *more and trying* ***riskier things****, getting* ***comfortable*** *with the idea of slitting my* ***wrists or overdosing. I’m going to die.*** | Reveals a deep focus on suicide, with many expressing intent to end their lives and referencing past struggles with suicidal thoughts and self-harm. |
| **Risk Factor: Thwarted Belongingness** | *I feel like a* ***real-life NPC****, unable to* ***connect*** *with my life despite having a stable living situation.* ***Childhood trauma****, particularly related to* ***homophobia****, may contribute to my* ***emotional numbness****. [..] Despite living in a* ***supportive environment*** *my* ***life feels pointless and detached****.* | Conveys deep emotional struggles, isolation, and detachment from life. |
| **Risk Factor: Feeling of Burdensomeness** | *I feel* ***useless and hypocritical****, struggling with* ***anxiety and depression****. I have* ***breakdowns and suicidal episodes*** *but feel guilty for asking for* ***help****. I resort to* ***cutting my forearms*** *to cope, torn between wanting to end my life and* ***longing for happiness****.* | Conveys deep feelings of worthlessness, guilt, and isolation. Individuals feel like a burden to their loved ones, intensifying their emotional pain and hopelessness. |
| **Risk Factor: Lethally Suicidal** | *I need help overcoming my* ***fear of suicide and death*** *because I've attempted* ***suicide twice but hesitated both times****. I feel like a* ***fraud*** *compared to those who go* ***through*** *with it, and when I tried not to eat as a* ***suicide attempt, I failed****.* | Reveals deep struggle with hopelessness, worthlessness, and isolation, often with a history of suicide attempts. They experience chronic loneliness and emotional disconnection from others. |

Table S8. Normalized distribution of BERT topics and themes across Dimension and RiskFactor.

| **Topic Theme** | **Lethally Suicidal** | **Thwarted Belongingness** | **Perceived Burdensomeness** | **Acquired Capability for Suicide** | **Lack of Reciprocal Love** | **Loneliness** | **Self-Hate** | **Liability** |
| --- | --- | --- | --- | --- | --- | --- | --- | --- |
| **Despair and Emotional Struggle** | 0.0119 | 0.0059 | 0.0145 | 0.0106 | 0.0061 | 0.0017 | 0.0097 | 0.0034 |
| **Substance Use** | 0.7016 | 0.6816 | 0.7167 | 0.6513 | 0.7147 | 0.1120 | 0.0774 | 0.0815 |
| **Seeking Support or Validation** | 0.1379 | 0.1576 | 0.1363 | 0.1524 | 0.1299 | 0.0480 | 0.0102 | 0.0226 |
| **Weakness and Pain** | 0.0007 | 0.0001 | 0.0003 | 0.0005 | 0.0002 | 0.0002 | 0.0003 | 0.0001 |
| **Planning and Attempts** | 0.0711 | 0.0570 | 0.0552 | 0.0815 | 0.0697 | 0.0186 | 0.0161 | 0.0286 |
| **Non**  **suicidal Self-injury (NSSI)** | 0.0029 | 0.0027 | 0.0021 | 0.0027 | 0.0023 | 0.0014 | 0.0020 | 0.0011 |
| **Methods and Tools** | 0.0895 | 0.0793 | 0.0706 | 0.0989 | 0.0718 | 0.0157 | 0.0056 | 0.0089 |
| **Cynicism and Bitterness** | 0.0040 | 0.0026 | 0.0058 | 0.0069 | 0.0060 | 0.0072 | 0.0035 | 0.0006 |

***Section S9. Evaluating and Validating IPTS Dimensions classification.***

Loneliness Classification: On the test dataset, this classifier showed an accuracy of 93%. The corresponding performance metrics were Precision: 0.94, Recall: 0.90, F1-score: 0.92, and AUC-ROC: 0.96.

Lack of Reciprocal Love Classification: This classifier showed an accuracy of 95%. The corresponding performance metrics were Precision: 0.95, Recall: 0.92, F1-score: 0.94, and AUC-ROC: 0.95.

Self-Hate Classification: The performance metrics for this classifier were Precision: 0.85, Recall: 0.70, F1-score: 0.77, and AUC-ROC: 0.85.

Liability Classification: This classifier showed a test accuracy of 87%. The corresponding performance metrics were Precision: 0.88, Recall: 0.82, F1-score: 0.85, and AUC-ROC: 0.90. To further assess construct specificity, we conducted targeted validation using held-out examples from the LoST (Linguistic markers of low self-esteem) dataset, which captures self-referential low self-worth expressions conceptually closer to self-hate than general aggression. The Self-Hate classifier demonstrated higher similarity alignment with these self-directed negative expressions compared with unrelated toxic content, supporting that the model captures internalized negativity rather than general hostility. While no distant dataset perfectly operationalizes self-hate, this additional validation strengthens the construct alignment of our approach beyond semantic proximity alone. The iterative process was stopped when subsequent RAKE rounds produced no substantively new, theoretically valid terms, indicating convergence rather than uncontrolled expansion. The final codebook size for each Dimension is mentioned in Table S4.

Further, we evaluated the dataset for the initial distantly supervised binary classifier model using a linguistic equivalence test inspired by prior work [83]. We measured word embedding-based similarities between the posts classified under each Dimension and corresponding semantically relevant datasets to understand if the dataset used was the correct dataset to train such a classifier model or not.

The results demonstrated high similarity scores, indicating strong alignment between distant data and classified Dimension posts of SI: 80.4% for loneliness, 83.3% for lack of reciprocal love, 94.3% for self-hate, and 76.2% for liability. These results reinforced the effectiveness of the semantically relevant datasets used to train the distantly supervised binary classifier, capturing the linguistic characteristics of each Dimension.

Furthermore, to understand the final classification of posts in the four RiskFactor, we conducted an expert validation of 450 classified posts. For Dimensions, we selected 50 posts each for loneliness, lack of reciprocal love, self-hate, and liability. For RiskFactors, we examined 50 posts for thwarted belongingness, perceived burdensomeness, acquired capability for suicide, and finally, 50 posts for lethally suicidal tendencies.

This dataset sample was manually verified by two psychologist co-authors with extensive experience in social media analysis and mental health disclosure research. Guided by the IPTS, these experts manually annotated a subset of posts for both Dimension and RiskFactor categories to evaluate the reliability of our automated labeling framework. Overall, we observed a high level of agreement between manual and model-based assessments, indicating strong alignment between theoretical constructs and automated inferences. Among Dimension labels, agreement rates were 94% for loneliness, 94% for lack of reciprocal love, 94% for self-hate, and 88% for liability. For RiskFactor labels, match rates reached 88% for thwarted belongingness, 88% for perceived burdensomeness, 80% for acquired capability for suicide, and 74% for lethally suicidal posts.

To better understand the nature of disagreements, we report confusion matrix counts in Table S6. Across Dimensions and RiskFactors, disagreements were distributed between false positives and false negatives, with no systematic pattern of under-detection. For the lethally suicidal category in particular, disagreements included 8 false positives and 5 false negatives, indicating that the model more often over-identified posts as high-risk than failed to detect expert-identified lethal posts. This bias toward caution aligns with the framework’s intended use as a screening aid, where sensitivity is prioritized to minimize the risk of missing potentially severe cases. Although the lethality dimension exhibited somewhat lower agreement, this aligns with prior research highlighting the inherent subjectivity and contextual variability of lethality assessment in suicide risk modeling [166, 167].

Expressions of suicidal intent often depend on nuanced contextual cues, indirect language, and fluctuating emotional states, making consistent annotation challenging even among trained clinicians. To address this, our framework is designed to function as a theory-guided screening and interpretive aid rather than a diagnostic classifier. Its purpose is to surface posts that may warrant closer human attention based on IPTS-aligned linguistic indicators, not to determine an individual’s true clinical risk or intent. In line with this role, the system prioritizes recall over precision to reduce the likelihood of missing potentially high-risk posts, an approach consistent with ethical best practices in automated suicide risk detection and early-warning systems [168, 169].

Importantly, model outputs are probabilistic and pattern-based, reflecting the presence of language consistent with IPTS constructs rather than definitive evidence of suicidal behavior. All posts identified as “lethally suicidal” by the model were subsequently reviewed by human experts, providing an additional safeguard and reducing the risk of inappropriate interpretation or action based solely on automated predictions. Collectively, the high concordance between manual and automated labeling, combined with structured human oversight, underscores the robustness and interpretive reliability of our IPTS-based framework for large-scale research on suicide-related discourse while maintaining clear boundaries from clinical or diagnostic use.

***Section S10. Threshold for Labeling Posts with IPTS Dimensions and Riskfactors.*** We chose a similarity threshold of 0.60 for labeling a post to a particular dimension. This threshold was determined through iterative experimentation of trial and error to optimize label quality for each post, and also aligns with prior research adopting similar methodologies of language embedding-based similarities [117, 118]. As shown in Table S2, lowering the similarity threshold from 0.60 to 0.55 increases the number of posts assigned to all IPTS categories, whereas raising the threshold to 0.65 results in consistently fewer classified posts. This pattern reflects the expected trade-off between sensitivity and specificity: a more lenient threshold captures more potentially relevant posts, while a stricter threshold yields a more conservative subset with higher semantic similarity to the codebook phrases. Table S3 further demonstrates that, despite these changes in volume, a substantial proportion of posts remain consistently labeled across thresholds. The high overlap values indicate that the core set of posts identified within each IPTS category is relatively stable, suggesting that the classification approach is robust to moderate variations in the similarity cutoff.
